# Tissue-specific enhancer repression through molecular integration of cell signaling inputs

**DOI:** 10.1371/journal.pgen.1006718

**Published:** 2017-04-10

**Authors:** Luis Humberto Mojica-Vázquez, Mikhail H. Benetah, Aissette Baanannou, Sandra Bernat-Fabre, Bart Deplancke, David L. Cribbs, Henri-Marc Bourbon, Muriel Boube

**Affiliations:** 1 Centre de Biologie du Développement (CBD), Centre de Biologie Intégrative (CBI), Université Fédérale de Toulouse, UMR5547 CNRS/Université Paul Sabatier (UPS), Toulouse, France; 2 Laboratory of Systems Biology and Genetics, Institute of Bioengineering, School of Life Sciences, École Polytechnique Fédérale de Lausanne (EPFL), Lausanne, Switzerland; University of California Davis, UNITED STATES

## Abstract

*Drosophila* leg morphogenesis occurs under the control of a relatively well-known genetic cascade, which mobilizes both cell signaling pathways and tissue-specific transcription factors. However, their cross-regulatory interactions, deployed to refine leg patterning, remain poorly characterized at the gene expression level. Within the genetically interacting landscape that governs limb development, the *bric-à-brac2* (*bab2*) gene is required for distal leg segmentation. We have previously shown that the Distal-less (Dll) homeodomain and Rotund (Rn) zinc-finger activating transcription factors control limb-specific *bab2* expression by binding directly a single critical leg/antennal enhancer (LAE) within the *bric-à-brac* locus. By genetic and molecular analyses, we show here that the EGFR-responsive C15 homeodomain and the Notch-regulated Bowl zinc-finger transcription factors also interact directly with the LAE enhancer as a repressive duo. The appendage patterning gene *bab2* is the first identified direct target of the Bowl repressor, an Odd-skipped/Osr family member. Moreover, we show that C15 acts on LAE activity independently of its regular partner, the Aristaless homeoprotein. Instead, we find that C15 interacts physically with the Dll activator through contacts between their homeodomain and binds competitively with Dll to adjacent cognate sites on LAE, adding potential new layers of regulation by C15. Lastly, we show that C15 and Bowl activities regulate also *rn* expression. Our findings shed light on how the concerted action of two transcriptional repressors, in response to cell signaling inputs, shapes and refines gene expression along the limb proximo-distal axis in a timely manner.

## Author summary

Limb morphogenesis is controlled by a well-known genetic cascade, mobilizing both cell signaling and tissue-specific transcription factors (TFs). However, how their concerted action refines gene expression remains to be deciphered. It is thus crucial to understand how cell signaling inputs are integrated by transcriptional “enhancers”. The *Drosophila* leg provides a good paradigm to dissect the molecular mechanisms underlying gene regulation. Here, we used the *bric-a-brac*2 (*bab2*) gene as a model to study the integrated regulation of patterning genes implicated in tarsal segmentation. *bab2* expression in the leg primordium is dynamic and complex, going from initial broad distal expression to precisely positioned tarsal rings. By genetic and molecular analyses, we show here that the cell signaling-responding TFs C15 and Bowl interact directly with the limb-specific *bab2* enhancer as a repressive duo. Moreover, C15 acts independently of its partner Aristaless through physical interaction with the Dll activator. We propose that Dll induces early circular *bab2* expression pattern, then EGFR signaling-induced C15 in the distalmost cells competes with Dll for LAE binding and resolves *bab2* pattern as a ring. Taken together our data shed light on how the concerted action of a quartet of transcription factors reshapes gene expression during limb proximo-distal axis development.

## Introduction

In developing arthropod and vertebrate appendages, morphogen gradients play critical roles in instructing the spatially-restricted expression of patterning genes that mostly encode transcription factors (TFs) [1, 2]. However, how their expression domains are set up precisely and how their cross-regulation refines pattern layouts, remain to be deciphered, particularly by characterizing the *cis*-regulatory modules within the gene regulatory networks governing limb development.

The *Drosophila* leg provides a paradigm with which to tackle the issue of the molecular mechanisms underlying proximo-distal limb development [2–4]. The primordia of the adult appendages, the leg imaginal discs, originate from clusters of roughly 20–30 embryonic ectodermal cells which proliferate during the three larval stages. During the first and second instar larval stages, in response to antagonistic *wingless* (*wg*) and *decapentaplegic* (*dpp*) cell signaling pathways, the TF-encoding *Distal-less* (*Dll*), *dachshund* (*dac*) and *homothorax* (*hth*) genes are activated in broad concentric domains within the leg disc, that prefigure the distal, medial and proximal subdivisions of the adult appendage, respectively [3]. By the end of the third instar larval (L3) stage, the leg disc is highly folded presaging movements towards a tri-dimensional structure.

The distal portion of the adult leg comprises the tarsus (ts), which is divided into five segments (ts1-5, from proximal to distal), and the pretarsus (pt), which is characterized by a pair of terminal claws. Around the early L3 stage, ~72–80 hours (h) after egg laying (AEL), the combinatorial activities of the leg “gap” genes *Dll*, *dac* and *hth* induce the expression of the tarsal-specific *rotund* (*rn*), *bric-à-brac* (*bab*) complex (*bab1 and bab2*) and *Bar* complex (*BarH1* and *BarH2*) genes in distal circular domains [5–8]. At 80-82h AEL, secreted Wg and Dpp molecules jointly activate the epidermal growth factor receptor (EGFR) signaling pathway in distalmost pretarsal cells [5, 7]. In turn, EGFR cell signaling activates the expression of pretarsal patterning genes, including the homeobox genes *aristaless* (*al*), *dlim1* and *C15* (also known as *clawless*), and spatially extinguishes expression of tarsal patterning genes, including *bab* and *Bar* complex loci [5, 7–10]. As for leg gap genes, most pretarsal and tarsal patterning genes encode DNA-binding transcriptional regulators, expressed either in the very center of the leg disc (e.g., *C15*/*clawless* and *al*) or in concentric rings (e.g., *bab2*, *BarH1* and *rn*) [2, 4]. Lastly, at mid-late L3 stage, activation of the Notch (N) cell signaling pathway through the restricted expression of the Delta (Dl) and Serrate (Ser) ligands (in response to leg gap combinatorial activities) is required for formation of the flexible joint connecting each leg segment, through induction of a set of target genes, including the related genes *odd-skipped* (*odd*), *drumstick* (*drm*), *bowl* and *sister-of-bowl* (*sob*) [4, 11, 12].

*Dl* and *Ser* expression boundaries are maintained by a negative feedback loop between N signaling and the Lines/Bowl pathway [13]. In *Dl* expressing cells, the Lines protein is nuclear, leading to specific degradation of the Odd-family zinc-finger (ZF) protein Bowl [13–15]. In the adjacent joint cells, N signaling induces the expression of redundant Odd-family ZF proteins (Odd, Sob and Drm), which specifically re-localize Lines to the cytoplasm, thus preventing Bowl destabilization; consequently, Bowl TF accumulates in the nuclear compartment where it down-regulates *Dl* expression [13]. This feedback mechanism reinforces a Dl+/Dl− boundary, ensuring maintenance of N signaling through joint morphogenesis. Several genes required for distal leg segmentation have altered expression in *bowl* mutant cells, such as *bab2* [16], but none have been shown so far to be under the direct control of the Bowl transcription factor.

The *bric-à-brac* complex is formed by the duplicated paralogous genes *bab1* and *bab2*, encoding BTB/POZ domain TFs with overlapping functional roles in several developmental processes [17, 18]. While *bab1* is expressed similarly to *bab2*, only the latter is critically required for distal leg and antennal segmentation [18]. The *bab2* gene displays dynamic expression in a restricted proximo-distal (P-D) expression pattern in the distal leg and antennal primordia [17–20]. Initially expressed as a broad circle within the *Dll-*expressing distal domain at early-mid L3 stage, the *bab2* pattern in late-L3-stage leg and antennal discs resolves to four or two concentric rings, respectively [20]. Later on, at the pupal and adult stages, a P-D graded pattern is observed for each *bab2*-expressing ring, which is essential for ts2-5 and antennal (a) a3-5 segment joint formation [18].

While many DNA-binding factors have been identified, little is known about their molecular relationships within the gene regulation landscape governing limb formation along the P-D axis. To this aim, we previously identified within the *bric-à-brac* locus a single *cis*-regulatory module that reliably governs *bab2* expression in developing appendages, termed LAE for leg/antennal enhancer, and showed that the Distal-less homeodomain and Rotund zinc-finger TFs interact directly with discrete critical sites within the LAE enhancer to activate or up-regulate *bab2* in all or specifically in the proximalmost expressing cells, respectively, within the developing distal leg and antenna [19]. Here, we show that the C15 homeodomain and Bowl zinc-finger proteins act jointly by binding directly conserved LAE sequences, to restrict *bab2* expression within the developing leg, in response to cell signaling. We show that C15 competes with the Dll activator for LAE binding on neighboring cognate sites. Furthermore, instead of engaging direct partnership with the Aristaless homeoprotein, we find that C15 interacts physically with Dll through contacts between their homeodomain. Taken together with our previous data, this study provides a deeper understanding of how a transcriptional enhancer integrates diverse repressive cell signaling inputs produced along the proximo-distal axis of the developing limb.

## Results

### LAE enhancer repression along the leg proximo-distal axis through its conserved CR2 sequence

**Fig 1:**
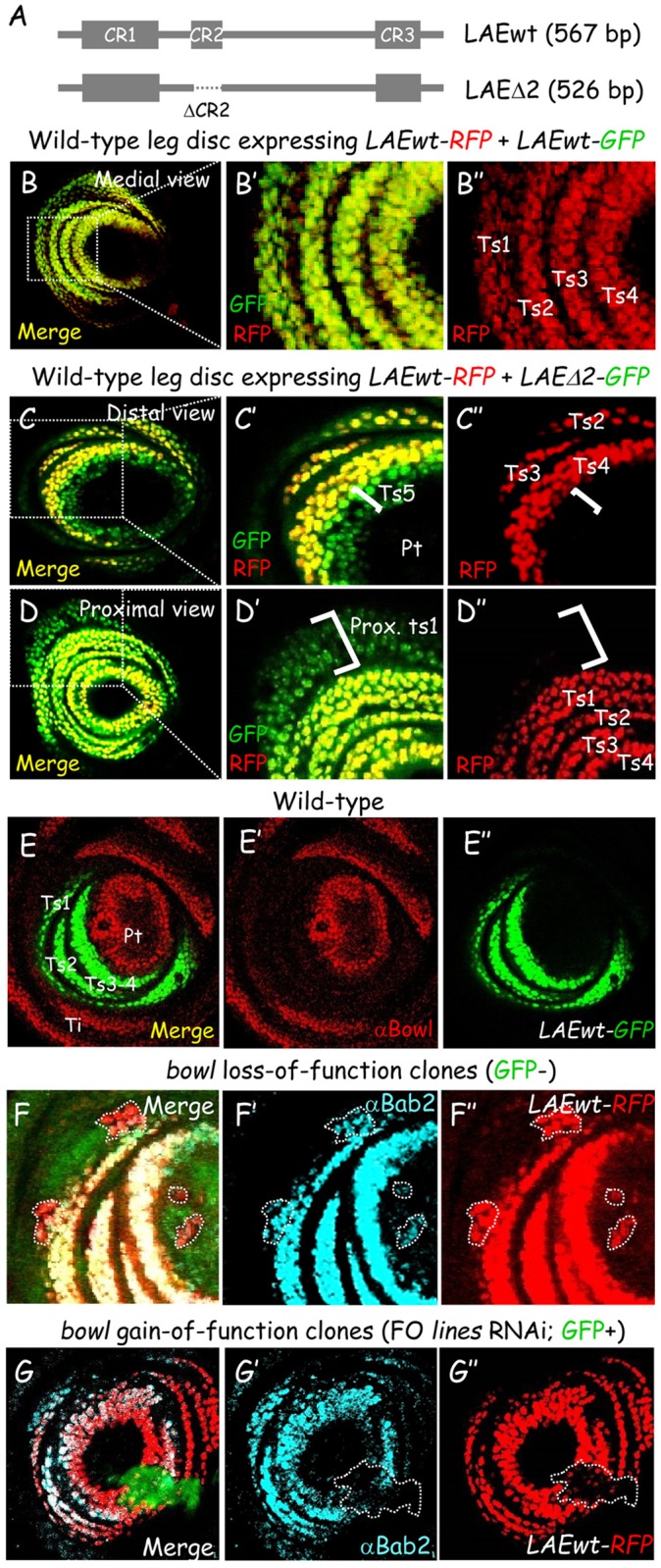
The Lines/Bowl pathway restricts LAE activity within the developing distal leg. (A) Scheme of the wild-type (wt) LAE enhancer and of its *Δ*CR2 derivative. Filled boxes indicate the three regions (CR1 to CR3) conserved among dipterans [19]. (B-D) Wild-type late L3 leg discs expressing *LAEwt-RFP*^*ZH*86Fb^ with either *LAEwt-GFP*^*ZH2A*^ (B; medial view) or *LAEΔ2-GFP*^*ZH2A*^ (C-D; distal and proximal views, respectively). Boxed areas are magnified for each confocal view, in (B’), (C’) and (D'), respectively, with a comparison to RFP fluorescence in isolation, in (B”), (C”) and (D”), respectively. Ectopic GFP expression at the distal (ts5) and proximal (ts1) edges of the *bab2*-expressing tarsal domain are indicated by white brackets. (E) Bowl expression within a wild-type late L3 leg disc expressing *LAE-GFP*. Merged Bowl immunostaining (red) and GFP fluorescence (green) is shown, as well as each of the two markers in isolation, in (E’) and (E”), respectively. (F-G) Mosaic late L3 leg discs expressing *LAEwt-RFP* and harboring either *bowl* null mutant clones (F) (detected by the loss of GFP) or *lines* dsRNA-expressing FO clones (G) (specifically expressing GFP). Merged GFP (green) fluorescence, LAE-driven RFP (red) fluorescence and Bab2 immunostaining (cyan) are shown, as well as Bab2 and RFP expression in isolation, in (F’, G’) and (F”, G”), respectively. Mitotic clones are circled with white dashed lines. Note that ectopic *bab2* (and *LAE-RFP*) expression is observed in *bowl* mutant clones both distally and proximally (F’, F”), and conversely their down-regulation is observed in Bowl-stabilized FO clones deficient for *lines* (G’, G”), in a cell-autonomous manner.

Limb-specific *bab2* expression is governed by a single 567-bp *cis*-regulatory module (CRM), termed LAE [19]. Among the three evolutionarily-conserved regions (CR1-3) comprised in this CRM, CR1 is critical for enhancer activity, notably through Dll activator binding [19]. Here, we have investigated the functional role of the 41-bp CR2 sequence. To this end, expression of an internally-deleted LAE construct (*LAEΔ2-GFP*) (Fig 1A) was compared to expression of a wild-type (wt) LAE reporter (*LAEwt-RFP*), faithfully reflecting the endogenous *bab2* gene [19]. Prominent ectopic expression from the *LAEΔ2-GFP* construct was detected at the proximal (ts1) and distal (ts5) edges of the RFP (*bab2*)-expressing tarsal domain (compare Fig 1B to 1C and 1D, white brackets), indicating a requirement for CR2 in defining *bab2* expression limits. However, GFP expression was absent from developing pretarsal cells, indicating that CR2 is dispensable for EGFR-signaling mediated *bab2* extinction there. We conclude that within the LAE enhancer, the conserved CR2 sequence is absolutely required for repressing LAE reporter (*bab2*) expression along the proximo-distal axis of the leg, to delimit precisely the tarsal expression borders.

### The Lines/Bowl pathway regulates LAE enhancer activity within the distal leg primordium

**Fig 2:**
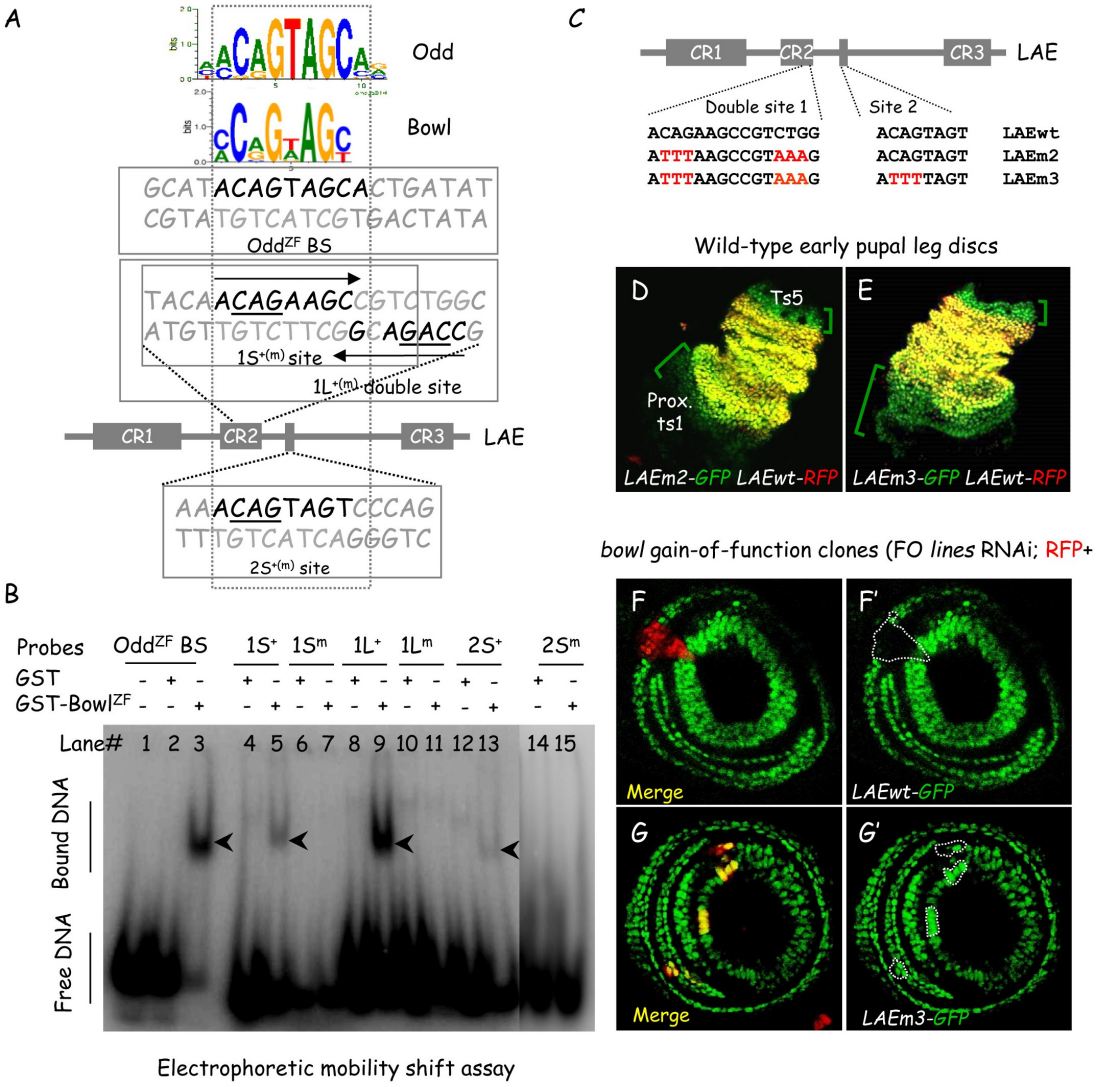
Bowl binds directly to LAE sequences *in vitro* to regulate LAE activity *in vivo*. (A) LOGOs of consensus Odd-family ZF binding sites [21] are shown in the upper part. The dotted line box indicates this consensus in the binding sites under investigation. The double-stranded sequences encompassing a *bona fide* Odd ZF-binding site [22] as well as the two consensus Bowl ZF-binding sites within the LAE are shown beneath. Corresponding probed double-stranded DNA sequences tested in EMSA are framed. The shared CAG sub-motifs in wild-type (+) sequences, substituted by TTT in each mutant (m) probe, are underlined. For the small (1S) and large (1L) CR2 probes, near palindromic sub-sequences are indicated by converging arrows. (B) EMSA experiments examining Bowl-DNA complexes. Each tested DNA probe, shown in (A), was incubated with either purified non-fused GST (as a negative control) or purified GST-Bowl^ZF^. Specific complexes are indicated by black arrowheads. Note that Bowl ZF binding to LAE sequences was fully abolished for each CAG mutant probe. (C) Wild-type as well as mutant LAE sequences encompassing the biochemically-defined Bowl ZF binding sites are shown, with mutated nucleotides in red. (D-E) Wild-type late L3 leg discs co-expressing *LAEwt-RFP* (red) and either *LAEm2-GFP* (D) or *LAEm3-GFP* (E) (green). Bowl ZF cognate binding sites are critical for restraining LAE reporter activity in distal ts5 and in proximal ts1. (F-G) Mosaic late L3 leg discs expressing either *LAEwt-GFP* (F) or *LAEm3-GFP* (G) and harboring FO clones (RFP+) expressing *lines* dsRNA (i.e., ectopically-stabilizing nuclear Bowl). Merged LAE-driven GFP (green) and FO RFP (red) fluorescence are shown, as well as GFP expression in isolation, in (F’) and (G’), respectively. Representative Clones deficient for *lines* activity are circled with white dashed lines. While ectopically-stabilized nuclear Bowl induced *LAEwt-GFP* repression (F’), it was unable to down-regulate the mutant LAE reporter (G’) (as revealed by the merged yellow instead of red color obtained in F).

The *lines*/*bowl* gene cassette has been proposed to regulate *bab2* expression along the leg P-D axis [13, 15, 16]. Indeed, tarsal *LAE-GFP* expression is flanked both proximally and distally by Bowl-expressing domains (Fig 1E). In contrast, *LAEΔ2-GFP* is up-regulated in Bowl-expressing cells at the ts5-pretarsal boundary (S1 Fig, compare panels A to B). Further, CR2 contains a consensus Bowl binding site (see Fig 2A). We thus tested the functional relationships between *bowl* and the LAE enhancer in loss- (lof) and gain-of-function (gof) clonal analyses in the leg disc. As for endogenous *bab2* [13], *LAEwt-RFP* expression was cell-autonomously de-repressed in *bowl*^-/-^ mitotic clones situated both proximally and distally within the developing distal leg (Fig 1F; clones are circled with white dashed lines). Ectopically-expressed Bowl protein accumulates poorly within nuclei of developing tarsal cells due to instability induced by nuclear Lines protein, a dedicated Bowl antagonist specifically expressed there [13, 16]. *bowl* gain-of-function was therefore obtained through RNA interference (RNAi) coupled to “flip out” (FO) Gal4 expression (see Materials and methods) to achieve clonal down-regulation of *lines* [13]. Endogenous *bab2* [13, 15] and *LAEwt-RFP* expression were both autonomously repressed in most *lines*-deficient FO cells (GFP+) generated within the developing leg (Fig 1G; a large clone is circled). Our data indicate that LAE enhancer activity is regulated by the Lines/Bowl pathway, raising the possibility that repressive Bowl transcription factor might bind directly to LAE sequences.

### Bowl zinc-finger protein binds directly conserved motifs within the LAE enhancer

Bowl belongs to the Odd family of zinc-finger TFs, and closely-related consensus DNA binding motifs have been recently defined for Odd [5’-(A/C)CAGTAGC] and Bowl [5’-(C/A)C(A/G)G(T/A)AG(C/T)] ZF domains by a bacterial one-hybrid approach [21]. Two sequences matching consensus binding sites (BS) for Odd/Bowl are present within the LAE enhancer (Fig 2A), and both are conserved among 24 available Drosophilidae *bric-à-brac* locus sequences (S2A and S2B Fig, respectively), one being precisely a part of the repressive CR2 sequence acting along the leg P-D axis (see Fig 1C and 1D). In addition to Drosophilidae species, the CR2 consensus site is conserved also in the Mediterranean fruit fly *Ceratitis capitata*, the tsetse fly *Glossina morsitans* and the domestic house fly *Musca domestica* (S2A Fig). Moreover, the putative Bowl BS in CR2 (5’-ACAGAAGC) is embedded within an imperfect palindromic sequence (5’-ACAGAAGCCGTCTGG; underlined nucleotides may constitute a second Bowl BS in an inverted orientation), which appears, however, to be strictly conserved only among Drosophilidae species (S2A Fig).

Full-length Bowl GST fusion is toxic when expressed in bacteria. To test for direct interaction with LAE sequences *in vitro*, we therefore expressed a 163 amino-acid long segment of the Bowl protein encompassing its zinc-finger domain (termed Bowl^ZF^) as a glutathione S-transferase (GST) fusion protein in *E*. *coli*. Purified GST-Bowl^ZF^ was examined for specific DNA binding in an electrophoretic gel mobility shift assay (EMSA) (Fig 2B). As a positive control, we used a DNA probe (Odd^ZF^ BS) previously shown to interact with a recombinant GST-Odd^ZF^ fusion protein [22]. DNA probes encompassing each consensus site within CR2 (1S+) and in the 3’-end neighboring conserved sequence (2S+) (see Fig 2A) interacted with GST-Bowl^ZF^, albeit weakly compared to the Odd ZF BS (Fig 2B, compare lanes 5 and 13 with lane 3), but not with unfused GST used as a negative control (lanes 2, 4 and 12). By contrast, GST-Bowl^ZF^ interacted strongly with a larger DNA probe (1L+) encompassing the entire CR2 near-palindromic sequence (compare panels 5 and 9), and binding was abolished when the common CAG nucleotides (underlined in Fig 2A) present in each probe were mutated to TTT (1Sm, 1Lm and 2Sm; lanes 7, 11 and 15, respectively). The shifted 1S+ and 1L+ DNA-protein complexes (black arrowheads) migrated similarly, suggesting that Bowl ZF fusion protein might interact as a monomer in both cases. Taken together, these data indicate that the Bowl ZF DNA-binding domain interacts specifically with dedicated LAE sequences *in vitro*, particularly with the near-palindromic site within CR2.

### The Lines/Bowl pathway regulates LAE enhancer activity through the repressive CR2 sequence

To evaluate whether the two conserved Bowl ZF-binding sites within the LAE enhancer are critical for *bab2* repression in the developing distal leg, we sequentially mutated the common CAG motif of the double site within CR2 and of the single one outside CR2 to TTT, to yield the LAEm2-GFP and then LAEm3-GFP derivatives (Fig 2C). When compared to the normal *bab2*-expressing tarsal domain (monitored with *LAEwt-RFP*), *LAEm2-GFP* displayed strong GFP de-repression in proximal ts1 and distal ts5 (Fig 2D), as previously seen for the CR2-deleted *LAEΔ2-GFP* reporter (above, Fig 1C and 1D). *LAEm3-GFP* (mutated for both Odd/Bowl ZF BS) was likewise de-repressed proximally and distally (Fig 2E). However, proximal *LAEm3-GFP* up-regulation is significantly broader, compared to *LAEm2-GFP* (compare the extents of the green brackets in Fig 2D and 2E), indicating that both conserved Bowl binding sites are required for full repression of the LAE enhancer in proximal ts1, while only the CR2 site is apparently needed in distal ts5.

To confirm that the Lines/Bowl pathway acts *in vivo* through the biochemically-defined binding sites within the LAE enhancer, we ectopically-stabilized nuclear Bowl protein in flip-out mitotic clones, by down-regulating *lines* activity within the developing leg through mosaic RNAi knockdown (above, see Fig 1G) and examining fluorescence patterns driven by thrice CAG-mutated *LAEm3-GFP*. Whereas the wild-type LAE reporter was repressed in *lines*-deficient tarsal cells (RFP+), *LAEm3-GFP* was impervious to repression by stabilized Bowl within the same cells (compare Fig 2F, 2F’, 2G and 2G’; *lines*-deficient FO clones are circled with white dashed lines). Therefore, we conclude that the Bowl transcription factor acts directly through its evolutionarily-conserved cognate binding sites within the LAE enhancer, to limit *bab2* expression in the developing tarsus.

### *C15*/*clawless* activity is required for *bab2* repression in the developing pretarsus

Previous work has shown that EGFR signaling extinguishes *bab2* expression within the distalmost subdivision of the developing leg, the pretarsus [5, 8]. The mutant LAE reporter *LAEm3-GFP*, while resistant to Bowl-mediated P-D repression (Fig 2G) is still repressed in the pretarsal primordium (Fig 2E), as visualized upon co-staining with the C15 specific marker (S1C Fig). Moreover, Bowl protein is expressed in a row of *C15*-expressing pretarsal cells at the ts5/pretarsal boundary, where *LAEm3-GFP* expression remains undetected (S1D Fig). Since LAE reporter expression is absent from *C15*-expressing cells, we asked whether C15 TF activity mediates EGFR signaling-dependent down-regulation of LAE enhancer activity in the developing pretarsus.

**Fig 3:**
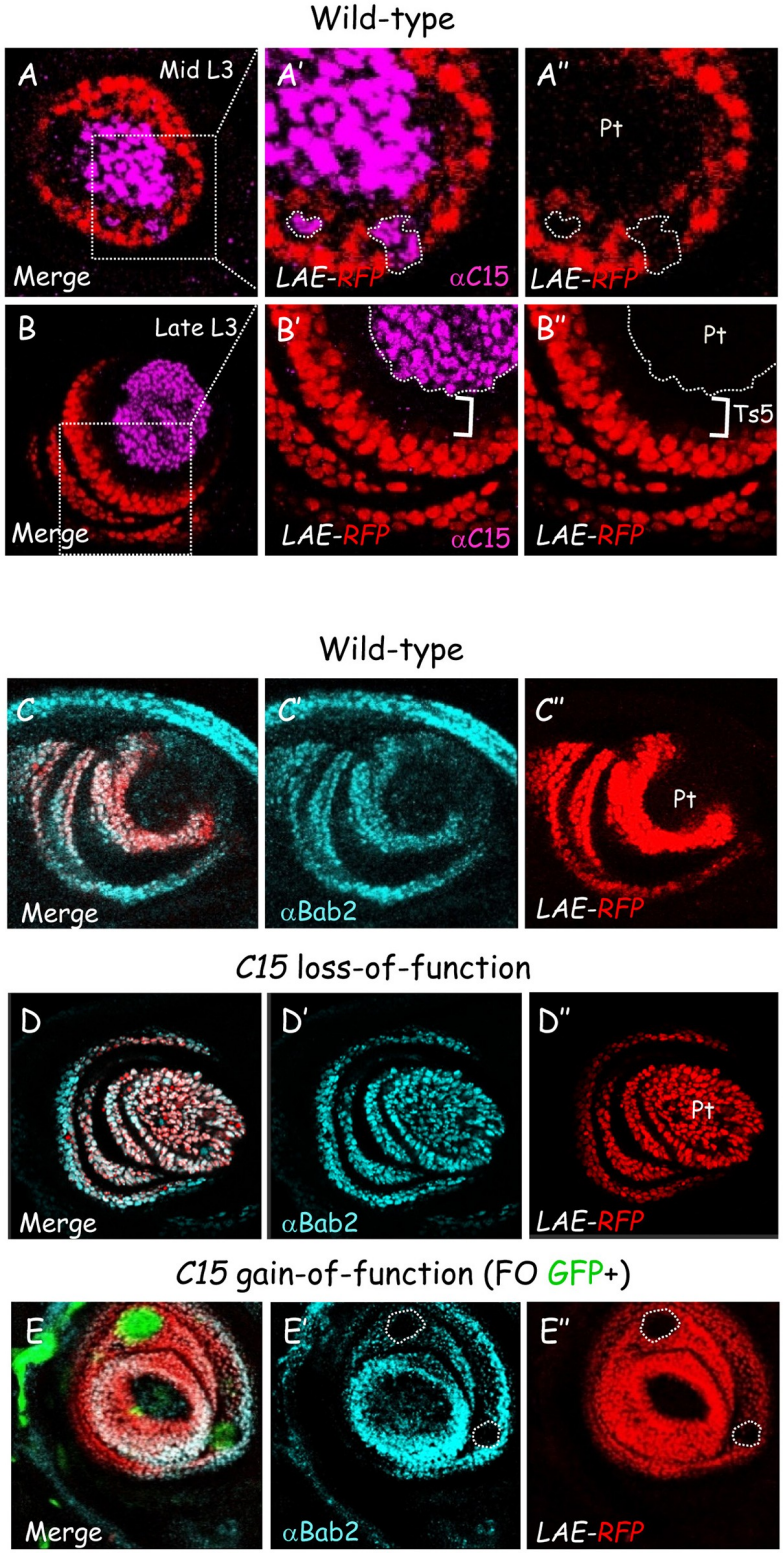
*C15* represses *bab2* and LAE enhancer activity in the developing leg. (A-B) Mid (A) and late (B) L3 leg discs expressing the *LAE-RFP* reporter. Merged LAE-driven RFP fluorescence (red) and C15 immunostaining (magenta) are shown. Boxed areas are magnified for each stage, in (A’) and (B’), in comparison with LAE-driven RFP expression in isolation, in (A”) and (B”), respectively. At mid L3 stage, *bab2* is expressed within a single tarsal cell ring abutting the *C15*-expressing pretarsal cells. Note that *LAE-RFP* expression is never detected in isolated peripheral *C15+* cells (circled with white dashed lines in A’ and A”). At late L3 stage, *C15* and *bab2* are no longer expressed in abutting territories but now separated by the ts5 primordium (white brackets in B’ and B”). (C-D) Wild–type (C) or *C15*^*2*^ homozygous mutant (D) late L3 leg discs expressing the *LAE*-*RFP* reporter. Merged Bab2 immunostaining (blue) and RFP fluorescence (red) are shown, as well as each marker in isolation, in (C’) and (D’) and (C”) and (D”). Note that *bab2* and *LAE-RFP* are both ectopically expressed in the mutant pretarsus, as well as in distal ts5. (E) Bab2 immunostaining (cyan) of a mosaic late L3 leg disc expressing *LAE-RFP* (red) and harboring FO clones (GFP+; green) ectopically-expressing C15 protein. Merged markers as well as *bab2* or *LAE-RFP* expression in isolation, in (E’) and (E”), are shown. Clones are circled with white dashed lines. A cell-autonomous down-regulation of both *bab2* and *LAE-RFP* is observed in tarsal cells ectopically expressing C15.

*C15* homozygous mutants for a null allele (*C15*^*2*^) survive to adulthood with distal leg defects, exemplified by the absence of claws [8]. In early-mid L3, *C15*/*clawless* expression is activated in the pretarsal primordium, in response to activation of EGFR signaling at the center of the leg disc, then subsequently maintained until the adult stage [8, 9]. To ask whether *C15* regulates LAE enhancer activity, we first compared expression of the *bab2* (*LAE-RFP* reporter) and *C15* genes in the leg disc. At ~84-90h AEL, *C15* and *LAE-RFP* are expressed in adjacent territories within the emerging tarsus/pretarsus (Fig 3A). Soon afterwards at mild-late L3 stage C15 and *LAE-RFP* expression domains become well separated from each other, through gradual restriction of the LAE reporter by repressive Bowl activity in developing distal ts5 cells (Fig 3B; white bracket) [16]. Note that in the mid-L3 leg disc, *LAE-RFP* reporter expression is never detected in C15+ cells split away from the emerging central *C15*-expressing domain (Fig 3A, circled areas; n = 20) (see also below), suggesting that C15 TF might be sufficient to cell-autonomously extinguish LAE activity in the developing pretarsus, as suggested by Campbell [8]. Consistent with this possibility, *bab2* (*LAE-RFP*) de-repression was observed in all pretarsal cells in *C15*^*2*^ homozygous late-L3 leg discs (Fig 3C and 3D). Surprisingly, in *C15* homozygous mutant larvae *bab2* (*LAE-RFP*) expression was likewise de-repressed in developing distal ts5 cells, where repressive Bowl activity should be acting given our previous data (see Fig 2D and 2E). In fact, distal *bowl* expression is fully lost in *C15*^*-/-*^ leg discs (compare S3A and S3B Fig) [8]. Altogether these data support the notion that the C15 transcription factor might mediate directly EGFR-induced *bab2* repression in developing pretarsal cells, in addition to allowing indirectly gradual repression in ts5 cells by stabilizing Bowl there through Notch signaling activation.

To examine whether *C15* activity is sufficient for *bab2* (*LAE-RFP*) down-regulation when ectopically expressed in developing tarsal cells, in accordance with a putative direct repression, we used the FO technique to misexpress C15 protein in the leg disc. Ectopic C15 expression led to cell-autonomous *bab2* (*LAE-RFP*) repression in tarsal FO clones (Fig 3E). Both the loss- and gain-of-function genetic experiments indicate that the C15 transcription factor down-regulates *bab2* and LAE enhancer activity within the developing pretarsus, and this negative regulation might occur possibly through direct interaction with LAE sequences.

### Repressive C15 and activating Dll homeodomain proteins interact with distinct LAE sequences

**Fig 4:**
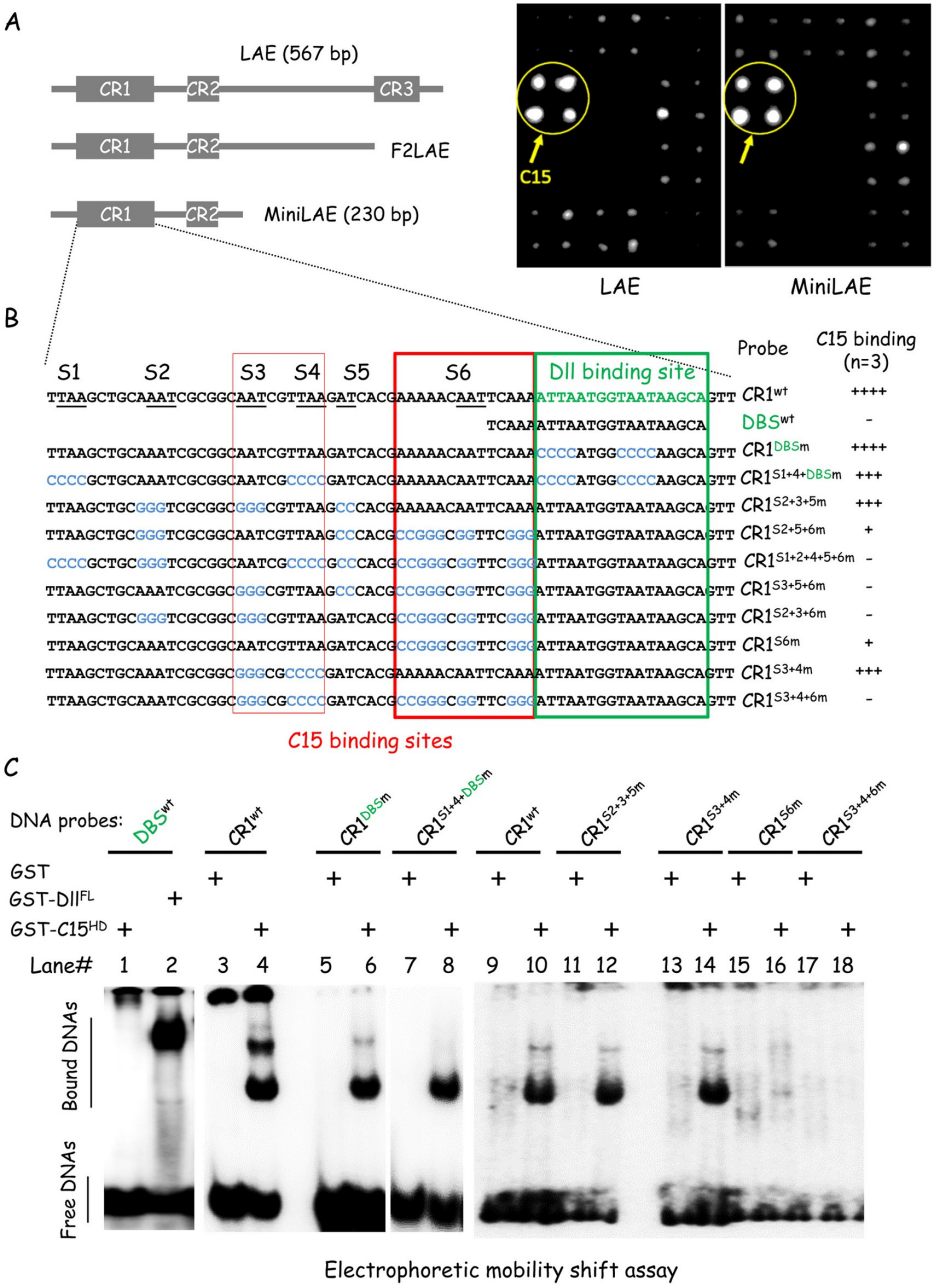
C15 homeoprotein interacts directly with LAE sequences. (A) C15 interacts with LAE in a yeast one-hybrid assay. Scheme of full-length LAE and of two shorter derivatives with slightly less (F2LAE) or much less (miniLAE) enhancer activities, are shown in the left part. The three evolutionarily-conserved LAE elements are indicated as grey-filled boxes. Examples of interactions are shown in the right with 12x4 yeast colonies expressing distinct *Drosophila* TFs fused to the Gal4 activating domain (AD) [23]. The quadruplicated colonies expressing the C15-Gal4AD fusion are circled, for both the entire and minimal LAE baits. TF-DNA bait interactions were identified based on growth on selective 20 mM 3-amino-1, 2, 4-triazole plates. Note that surrounding colonies are considered negatives and represent overall background growth. (B) Probing C15 HD binding to wild-type and mutant CR1 in EMSA experiments. Sequence of the evolutionarily conserved CR1 region is shown in the upper part. Dll-binding sub-region (DBS) is depicted in green and A/T-only sequence motifs (of at least two nucleotide long, and prone to interact with any homeodomain) are underlined. Mutated CR1 probes are shown beneath, with mutated nucleotides in blue. The Dll (DBS) and C15 binding sites are framed in green and red, respectively. The relative efficiency of C15 HD binding for each tested probes in shown on the right, quantified from 3 experiments. (C) Like Dll homeoprotein, purified C15 HD interacts with CR1 in EMSA. Tested probes are indicated above each lane. Purified GST was used as a negative control. Protein-DNA complexes from lanes #1–2, #3–8 and #9–18, respectively, were analyzed on three distinct native polyacrylamide gels.

The first indication for direct C15 binding to the LAE enhancer came from its identification as an LAE–interacting protein through a one-hybrid (Y1H) assay in yeast cells. Taking advantage of a nearly-complete *D*. *melanogaster* TF prey library (i.e., 692 upon 755 predicted DNA-binding regulators) [23], we performed a Y1H screen using as DNA bait the entire LAE or a 230 base pair (bp) 3’-truncated derivative (minimal CRM or miniLAE; see Fig 4A, left part). Note that miniLAE appeared sufficient (albeit with lower enhancer efficiency) for tissue-specific expression in a reporter assay, notably repression in pretarsal cells [19]. Significantly, from two independent Y1H screens, each done in duplicate, a strong TF-LAE interaction, corresponding to the C15 homeodomain protein (homeoprotein), reproducibly stood out for both constructs (Fig 4A, right part).

Given that Y1H assays reveal direct interactions, we sought to identify C15 binding site(s) [24, 25] within the miniLAE sequence (Fig 4B), that includes both the conserved CR1 region critical for activation, and the Bowl-interacting CR2 [19]. Neither the deletion of CR2 (above, Fig 1C and S1B Fig) nor systematic mutagenesis of CR1 sub-motifs [19] resulted in LAE-driven reporter gene de-repression in developing pretarsal cells. Thus, repressive C15 homeoprotein TF might act through redundant homeodomain (HD) binding sites or, alternatively, compete with activating Distal-less homeoprotein for shared HD cognate sites within CR1 [19]. The 69-bp CR1 sequence is highly enriched in A/T nucleotides (67%; Fig 4B), including the previously-identified Dll-binding sequence (DBS) within its 3’-moiety (a 22-bp region encompassing three canonical TAAT homeodomain binding sites) [19]. Using a purified recombinant GST fusion protein, we asked whether a 101 amino-acid-long C15 fragment encompassing the homeodomain (C15^HD^) can bind stably the DBS in a gel retardation assay. Contrary to a purified GST-Dll fusion used as a positive control, GST-C15^HD^ did not bind detectably the DBS probe (Fig 4C, lanes 1–2). In striking contrast, GST-C15^HD^ bound strongly a DNA probe encompassing the whole CR1 sequence, forming up to three protein-DNA complexes depending on EMSA conditions (Fig 4C, lanes 3–4). The simplest interpretation is that several GST-C15^HD^ molecules can interact simultaneously with several cognate binding sites distinct from the Dll DBS within CR1. To test this possibility, we mutated the Dll DBS TAAT sites, as reported in [19]. The GST-C15^HD^ fusion readily interacted with mutated CR1 (CR1^DBSm^), leading to equivalent retarded protein-DNA complexes (Fig 4C, lane 6; quantification from three experiments is shown in Fig 4B, on the right side). This result suggested that C15 homeodomain interacts mainly with sequences outside the DBS. Next, we sought to identify C15^HD^ binding sequences among the numerous A/T-rich motifs (prone to interact with C15 HD) present within CR1 (termed S1 to S6; underlined in Fig 4B). In addition to the TAAT sequences within the DBS, we tested a probe (CR1^S1+4+DBSm^) also mutated for the non-consensus TAA sequences within the S1 and S4 motifs. The GST-C15^HD^ fusion still interacted with CR1^S1+4+DBSm^ (Fig 4C, lane 8), albeit with slightly lower efficiency (see Fig 4B, right side) and with only one detectable retarded complex (Fig 4C, compare lanes 4 and 8). Second, we tested a series of probes mutated for at least three A/T motifs (formed by at least two consecutive A or T), substituting them by purely G/C sequences (Fig 4B; depicted in blue), leaving intact the DBS. Binding was faint for all probes mutated for S6, or absent when S3 or S4 were also mutated (Fig 4C and S4A Fig), suggesting that S6 and S3-4 are respectively high and low affinity cognate sites. We then tested a second series of probes specifically mutated for S3-4 (CR1^S3+4m^), S6 (CR1^S6m^) or both (CR1^S3+4+6m^) (see Fig 4B for sequences). As expected, GST-C15^HD^ interaction was slightly reduced (CR1^S3+4m^), strongly reduced (CR1^S6m^) or abolished (CR1^S3+4+6m^) (Fig 4C, lanes 14, 16 and 18, respectively; see Fig 4B for quantification). Altogether, these biochemical data indicate that C15 homeodomain interacts strongly with S6 and much less efficiently with S3-4 binding sites within CR1.

We then asked whether the C15 HD binding sites within the critical 69-bp CR1 region contributes to LAE repression *in vivo*. Given that systematic linker scanning mutagenesis of CR1 never resulted in de-repression [19], transgenic lines expressing *LAE-RFP* derivatives mutated for both the major (S6) and minor (S3-4) sites were generated. In contrast to a wild-type *LAE-RFP* construct inserted at a same genomic site, none of the two tested mutated derivatives (miniLAE and F2LAE, a larger form without the TAA-rich CR3 prone to interact with homeoproteins; Fig 4A and [19]) was detectably expressed within the leg disc (S4B Fig). These results suggest that in addition to a role in C15-mediated repression S3-4 and/or S6 CR1 sequences are also required for enhancer activation in the developing leg, which thus preclude from evaluating their implication in distal repression. Consistent with this hypothesis, our previous linker scanning mutagenesis revealed partially-redundant activating regulatory information within CR1, notably for subsequences encompassing the C15 binding sites [19].

Although C15 binding remains to be formally established *in vivo*, our data suggest that the EGFR-responsive C15 transcriptional repressor extinguishes *bab2* in the developing pretarsus by interacting directly with the LAE enhancer, at least through two cognate binding sites within CR1.

### C15 represses LAE activity on its own, without functional partnership with aristaless

C15 has been shown to function together with Aristaless (Al), another homeoprotein [9]. Al and C15 proteins are co-expressed in the developing pretarsus and both HD proteins interact cooperatively with a composite DNA binding site [(T/C)TAATTAA(T/A)(T/A)G][26]. However, the assumption of a systematic partnership between C15 and Al is based on the single identified common target locus *Bar*. Given that C15 HD binds directly LAE sequences distinct from the *Bar* enhancer (i.e. without TAATTAA core sequence; see Fig 4B), we next asked whether C15 acts independently of Al in repressing LAE activity.

**Fig 5:**
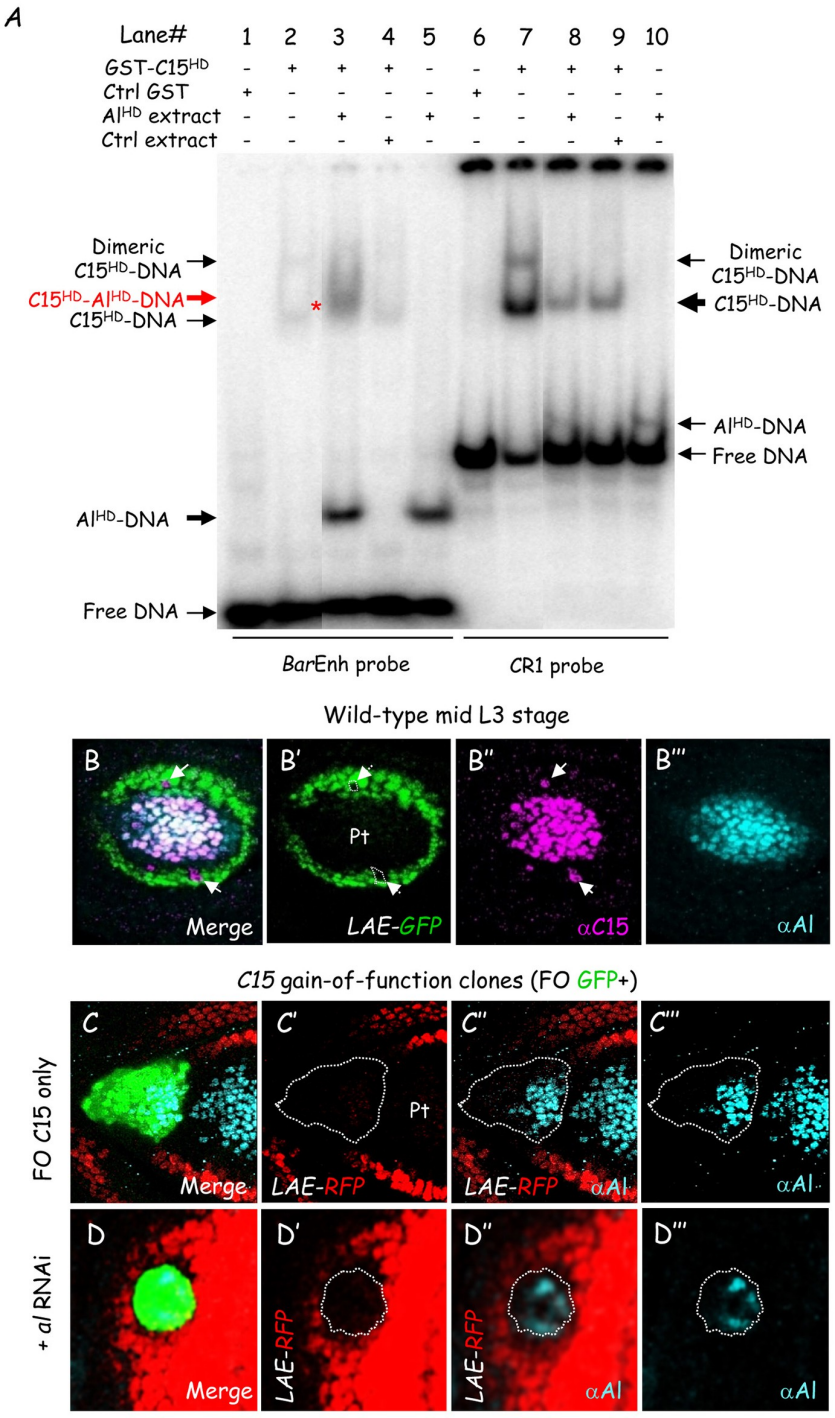
C15 functions independently of aristaless. (A) DNA-binding interaction between purified recombinant GST-C15^HD^ and *in vitro* translated Al HD on *Bar* and *bab2* enhancer sequences was examined in EMSA. In striking contrast to the BarEnh (compare lanes 2 and 3), upon addition of Al^HD^ no heterodimeric C15-Al DNA complex (red asterisk in lane 3) could be detected with the CR1 probe (compare lanes 7 and 8). Note that adding control reticulocyte extracts led to the same diminished GST-C15^HD^ binding to the CR1 probe. (B) Wild-type mid L3 leg disc expressing *LAE-GFP*. Merged GFP fluorescence (green) together with C15 (magenta) and Al (blue) immunostainings is shown, as well as each markers in isolation in (B’), (B”) and (B”‘), respectively. Note that isolated C15+ cells neither expressed *LAE-RFP* nor Al. (C-D) Mosaic Late L3 leg discs expressing the *LAE-RFP* reporter and harboring FO clones ectopically-expressing C15 either alone (C) or together with *al* dsRNA (D). Merged GFP fluorescence (green), RFP fluorescence (red) and Al immunostaining (cyan) are shown, as well as RFP and Al together, in (C”) and (D”), and both signals in isolation, in (C’) and (C”‘) and in (D’) and (D”‘), respectively. Despite Al depletion in a majority of C15-misexpressing FO cells, *LAE-RFP* expression remained fully down-regulated within all the cells of the examined clones (n = 8).

First, we tested whether C15 binds cooperatively with Al to LAE sequences *in vitro*. Given that C15 and Al interact physically through contacts between their homeodomain [26], we performed EMSA experiments with protein fragments sufficient for intermolecular interaction. Purified GST-C15^HD^ (see above) was combined with *in vitro* translated Al homeodomain (Al^HD^; Fig 5A). As a positive control, we used the *Bar* enhancer probe (BarEnh). In accordance with previous data [9], while GST-C15^HD^ bound poorly to the BarEnh probe, a new retarded complex was clearly observed upon addition of Al^HD^ extracts (Fig 5A, compare lanes 2 and 3). In striking contrast, GST-C15^HD^ bound strongly to the CR1 probe alone and adding Al^HD^-containing extracts did no yield heterodimeric complex (lanes 7–8). In fact, C15 HD binding was not enhanced, but instead diminished presumably due to the presence of competitors in the reticulocyte extracts (compare lanes 8–9). While Al homeodomain bound strongly to the BarEnh probe, it interacted poorly with the CR1 probe (compare lanes 5 and 10). Altogether these data indicate that C15 HD interacts strongly *in vitro* with LAE sequences independently of Al.

Second, we asked whether C15 homeoprotein is able to down-regulate LAE activity independently of Al *in vivo*. A first indication came from the observation in early-mid L3 that *LAE-RFP* expression is never detected in C15+ cells split away from the central domain (above, see Fig 3A), suggesting a cell autonomous C15-mediated repression. We found that those peripheral C15+ cells never co-expressed Al (n = 15) (Fig 5B, see white arrows), indicating that this repression does not require Al activity. A second indication came from C15 mis-expression within the *bab2*-expressing tarsal field. We previously showed that ectopic C15 expression in FO clones is sufficient to down-regulate LAE activity within the developing tarsus (above, see Fig 3E). *C15* ectopic expression in tarsal FO clones has been shown in fact to be sufficient to induce *al* expression in a cell autonomous manner [8], although not in all the C15-misexpressing cells (Fig 5C). To separate the roles of C15 and Al in LAE-RFP repression, we used the FO technique to couple C15 gain-of-function with RNAi-induced Al knock-down. Although dsRNA treatment was not sufficient for *al* extinction everywhere, an autonomous *LAE-RFP* down-regulation was still observed in all C15+ cells, even those without detectable Al protein (Fig 5D). Altogether these data provide evidence that C15 transcription factor represses directly LAE activity *in vivo* independently of Al.

### C15 and Dll homeoproteins compete *in vitro* and *in vivo*

**Fig 6:**
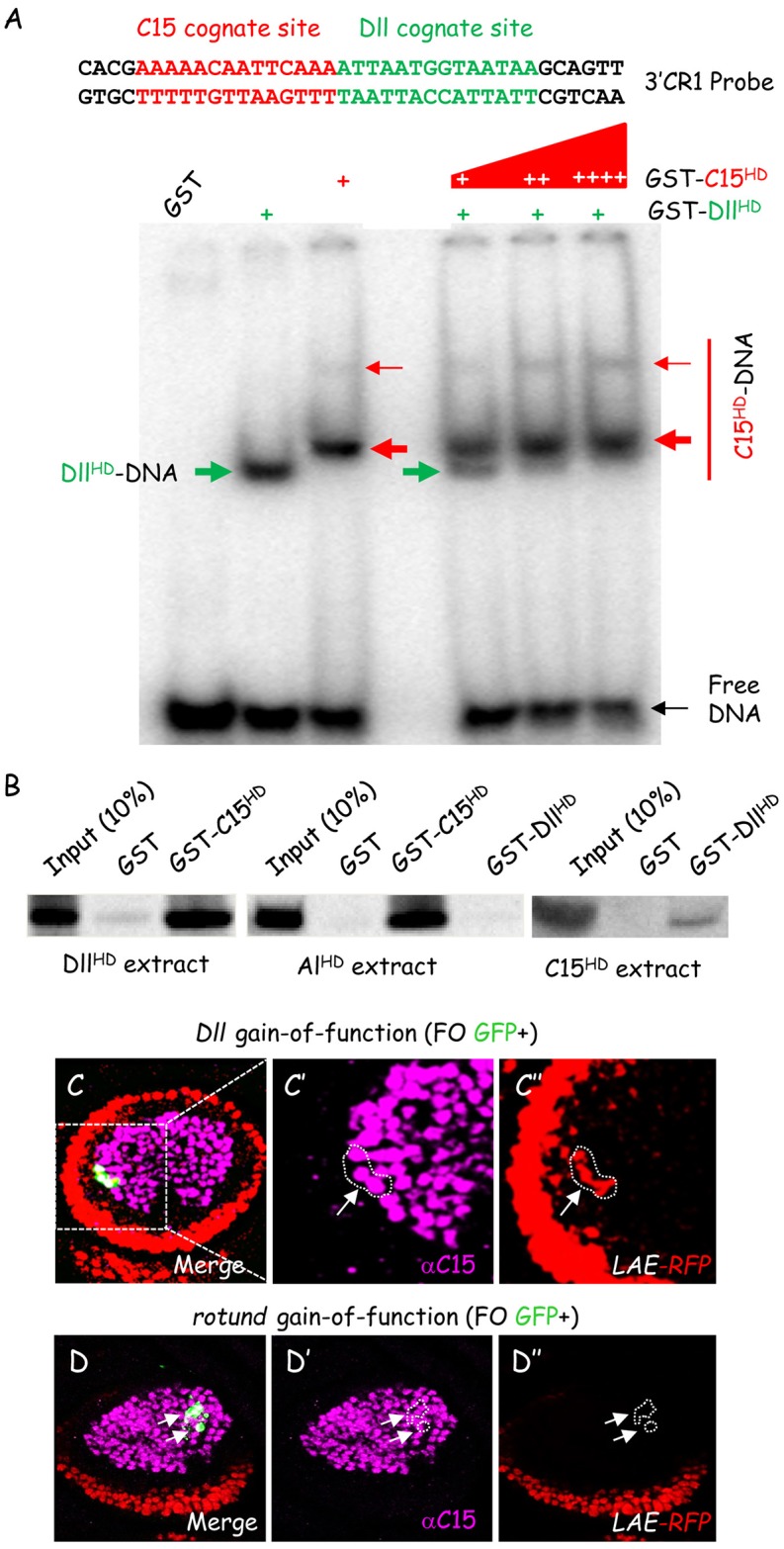
C15 and Dll interact functionally. (A) C15 and Dll outcompete for CR1 binding. EMSA was done with a probe encompassing the DBS and the neighboring S6 C15 binding site (CR1 3’ half; upper panel). Dll^HD^ and C15^HD^ GST fusions were tested in isolation or together with increasing amount of purified GST-C15^HD^. No heterodimeric C15-Al DNA complex could be detected. Moreover, GST-Dll^HD^ binding was abolished in a dose-dependent manner in favor of GST-C15^HD^. (B) Pull-down experiments with purified GST-C15^HD^ or GST-Dll^HD^ fusions and *in vitro* translated C15, Dll or Al HD. (C-D) Mosaic late L3 leg discs expressing the *LAE-RFP* reporter and harboring FO clones (GFP positive) (circled with white dashed lines) misexpressing C15 (C) or Rn (D) proteins. Merged LAE-driven RFP fluorescence (red) and C15 immunostaining (magenta) are shown, as well as each marker in isolation, in (C’–C”) and (D’-D”), respectively. Note that *LAE-RFP is* specifically de-repressed in Dll overexpressing FO clones without detectable change in C15 expression.

Repressive C15 and activating Dll homeoproteins bind strongly adjacent cognate sites within CR1 (above, Fig 4B). We next asked whether they can do so simultaneously. To this end, we tested their binding with a probe encompassing the high-affinity C15 as well as the neighboring Dll DBS sites (corresponding to the 3’ half of CR1; Fig 6A, see upper part). In addition to the GST-C15^HD^ protein described above, we purified a Dll fusion protein (GST-Dll^HD^) comprising only its homeodomain. As expected each homeodomain fusion bound the 3’ CR1 probe (Fig 6A, left lower part), yielding either a strictly monomeric GST-Dll^HD^ or a monomeric as well as some detectable dimeric GST-C15^HD^ complexes. To ask whether C15 and Dll homeodomains can interact jointly with their respective CR1 binding sequence, we simultaneously added the same amount of proteins in the DNA retardation assay. Significantly, no heterodimeric complexes could be detected, and respective monomeric complexes were even diminished. Moreover, adding increasing amounts of GST-C15^HD^ resulted in disappearance of GST-Dll^HD^ bound probe in favor of bound GST-C15^HD^ (Fig 6A, right lower part). Thus C15 and Dll compete for CR1 binding *in vitro*.

C15 and Al interact physically through contacts between their homeodomain [26]. We therefore asked whether C15 also establishes intermolecular interactions with the Dll HD, by performing pull-down experiments with purified GST-C15^HD^ and GST-Dll^HD^ fusions. *In vitro* translated ^35^S-labelled Dll homeodomain interacted with GST-C15^HD^ as efficiently as Al HD (Fig 6B, compare middle to left panels). Likewise, *in vitro* translated ^35^S-C15 homeodomain bound to GST-Dll^HD^ in the reciprocal test (right panel). Significantly, GST-Dll^HD^ did not interact with radiolabeled Al homeodomain. Thus, Dll and C15 engage specific protein-protein interaction via their respective DNA-binding domain, providing a possible rationale for their competitive interaction with LAE sequences.

Lastly, we examined the functional antagonism of C15 and Dll *in vivo*. We postulated that the down-regulation of LAE expression observed on ectopically expressing C15 in developing tarsal cells (above, Fig 3E), involves direct competition with activating Dll. Accordingly, we asked whether Dll up-regulation in pretarsal cells could impede repression by C15. On overexpressing Dll activator in C15+ FO clones (that are likewise Dll+), clear de novo *LAE-RFP* expression could be detected in all pretarsal clones (n = 12) (Fig 6C, see white arrow), while *C15* expression remained apparently unchanged (Fig 6C’). These data indicate that increased Dll protein is able to specifically counteract C15 repressive activity in pretarsal cells. Importantly, *LAE-RFP* up-regulation was never observed on ectopically expressing Rn protein (Fig 6D and see also below), indicating that the Dll-induced de-repressive effect is not a general effect of *bab2* activators. Given that C15 and Rn interact directly with well separated LAE sequences [19], the functional specificity of up-regulated Dll on C15 repressive activity is consistent with their binding site proximity.

Taken together, these data provide evidence for a new layer of *bab2* regulation by C15, through direct physical interaction with the Dll activator and competitive binding to neighboring cognate sequences within the LAE. In fact, three non-exclusive distinct mechanisms can be envisioned for C15 repressive activity: (1) active repression due to direct C15 binding to CR1 sequences, (2) steric hindrance toward Dll activator binding to adjacent cognate sites and (3) heterodimerization with Dll changing its binding specificity.

### C15 and Bowl proteins act jointly as a repressive TF duo responding to cell signaling

**Fig 7:**
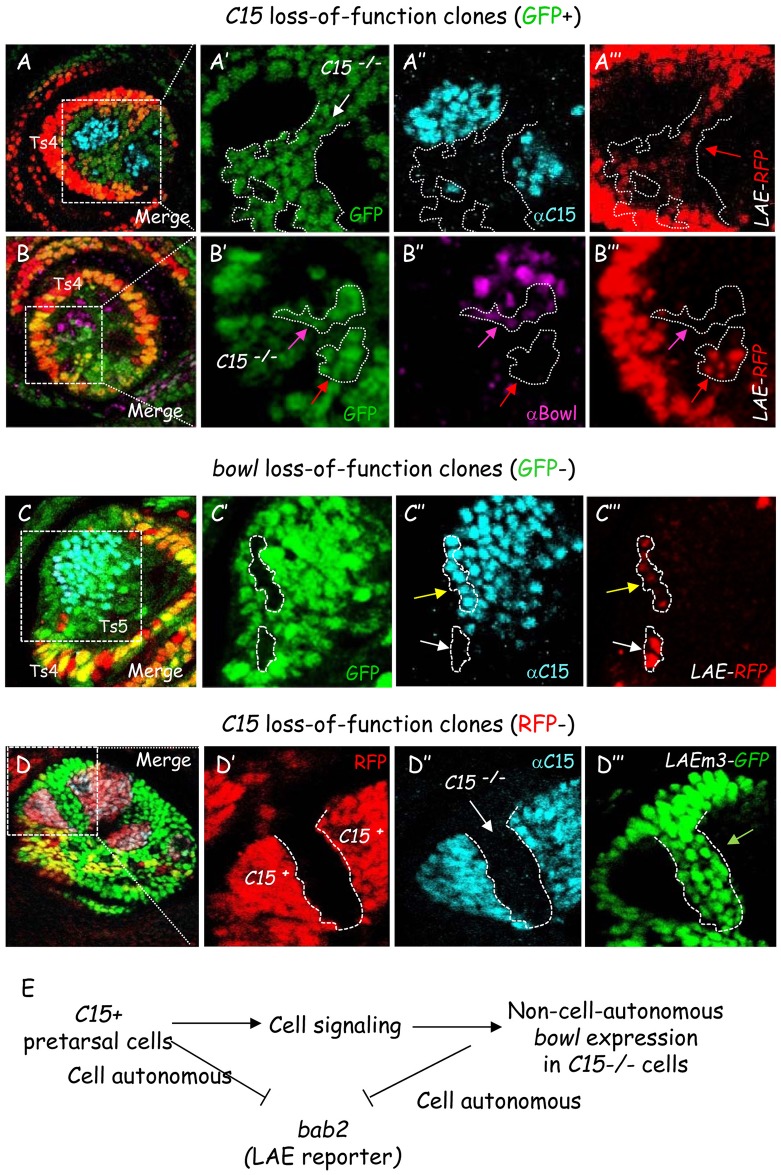
*C15* down-regulates LAE in the developing pretarsus, cell autonomously as well as non-autonomously through Bowl stabilization. (A-B) Mosaic late L3 leg discs harboring *C15* loss-of-function clones (GFP positive) in the presence of the *LAEwt-RFP* reporter. Merged GFP (green) and LAE-driven RFP (red) fluorescence together with C15 (cyan) (A) or Bowl (magenta) (B) immunostaining are shown. Boxed distalmost areas are magnified for each marker in isolation, in (A’, B’), (A”, B”) and (A”‘, B”‘), respectively. A faint *LAEwt-RFP* up-regulation (red arrow) is observed in mutant pretarsal cells (white arrow) situated farthest away from *C15+* (GFP negative) cells, i.e., black areas in (A’) and (B’). Strongest *LAEwt-RFP* up-regulation is observed in *C15* mutant cells without detectable nuclear Bowl (red arrows). Conversely, *LAEwt-RFP* expression is not observed in *C15*^*-/-*^ cells expressing Bowl protein (magenta arrows). (C) Mosaic late L3 leg disc expressing *LAE-RFP* and harboring *bowl*^*-/-*^ clones (GFP-; i.e. black areas). Merged GFP (green), C15 (cyan) and LAE-driven RFP (red) expression is shown. The boxed distalmost area is magnified for each marker in isolation, in (C’), (C”) and (C’”), respectively. *LAEwt-RFP* is strongly de-repressed in *bowl* mutant cells which does not express *C15* (white arrow), whereas only a faint up-regulation is observed in pure pretarsal clones. Note that *bowl* activity is not required for *C15* expression there. Taken together, this result indicates that C15 TF activity is not sufficient for full LAE repression in the developing pretarsus. (D) Mosaic late L3 leg disc harboring *C15*^*-/-*^ clones (detected by the absence of RFP fluorescence; i.e., black areas are mutant for *C15* here; see Materials and methods), in the presence of the *LAEm3-GFP* reporter. Merged RFP (red), C15 (cyan) and LAE-driven GFP (green) expression is shown. The boxed distalmost area is magnified for each marker in isolation, in (D’), (D”) and (D”‘), respectively. Note that full de-repression of Bowl-unresponsive *LAEm3-GFP* reporter is now observed (green arrow in D”‘) in most if not all pretarsal cells of a large *C15* mutant clone (white arrow in D”), to reach expression levels equivalent to those observed in neighboring tarsal cells. (E) Epistatic relationships between *C15* and *bowl* and their joint repressive activity on *bab2* (*LAE-RFP*) expression through cell autonomous and non-autonomous effects.

Surprisingly, while in *C15*^*2*^ homozygous leg discs we observed a clear ectopic *bab2* expression in all cells of the developing mutant pretarsus (above, Fig 3D), only a faint de-repression of *LAE-RFP* was detected within mutant clones (Fig 7A). Moreover, *LAE-RFP* de-repression only occurred in mutant cell subsets. This localized slight de-repression (red arrow) corresponded to those mutant cells situated farthest away from remaining *C15*+ cells (as detected by the presence of C15 antibody staining as well as absence of GFP fluorescence). This observation suggested that in addition to its cell-autonomous repressive activity, *C15* could also activate non-autonomously another *bab2* repressor. As mentioned above, *C15* induces *bowl* expression in a non-cell autonomous manner through N signaling activation [8]. We therefore asked whether Bowl TF could be responsible for this non-autonomous repressive effect. As shown in Fig 7B, Bowl protein was indeed expressed (as detected by the presence of Bowl antibody staining) in some *C15* mutant clones (expressing GFP) surrounding remaining C15+ cells (GFP-). Furthermore, *LAE-RFP* expression was detected only in *C15*^*-/-*^ cells (n>50) that corresponded precisely to those that lacked detectable Bowl protein accumulation (Fig 7B, red arrows). This observation suggested that in *C15* mutant clones even low amounts of Bowl protein (magenta arrows) are sufficient to fully extinguish LAE reporter activity. In support of this, in mosaic leg discs harboring *bowl* loss-of-function clones a faint *LAE-RFP* de-repression was observed in all *bowl*^*-/-*^ clones situated within the C15-expressing pretarsal field (Fig 7C, see yellow arrow). Moreover, the developing pretarsus does not detectably accumulate nuclear Bowl TF except for a single row of cells at the ts5-pt boundary (see S1B Fig).

To explore the hypothesis of Bowl-mediated C15 dependent repression, we then performed *C15* clonal loss-of-function analysis with *LAEm3-GFP*, specifically refractory to Bowl repressive activity (above). Note that in this experiment, wild-type cells (as detected with C15 immunostaining) are marked with RFP. In this effectively Bowl-inoperative context, *LAEm3-GFP* expression was fully de-repressed (green arrow) in most if not all *C15*^*-/-*^ pretarsal cells (visualized here by the absence of RFP fluorescence) (Fig 7D), indicating that the previously-observed non-autonomous repressive effect induced by C15^+^ cells is no longer at work. Taken together, we conclude that C15 homeodomain and Bowl zinc-finger proteins act as a repressive TF duo by repressing directly LAE enhancer activity in the developing leg, in response to a cell signaling cascade, to refine the distal *bab2* expression border.

### Epistatic relationships between *bab2* regulators

Together with the Dll and Rn activators, Bowl and C15 repressors constitute a TF quartet binding directly discrete sequences within the LAE enhancer, to ensure dynamic resolution of *bab2* expression in the developing distal leg. We next asked whether they engage cross-regulatory interactions. Contrary to *Dll*, transcriptional enhancers have not been yet characterized for *rn*, *bowl* and *C15*.

**Fig 8:**
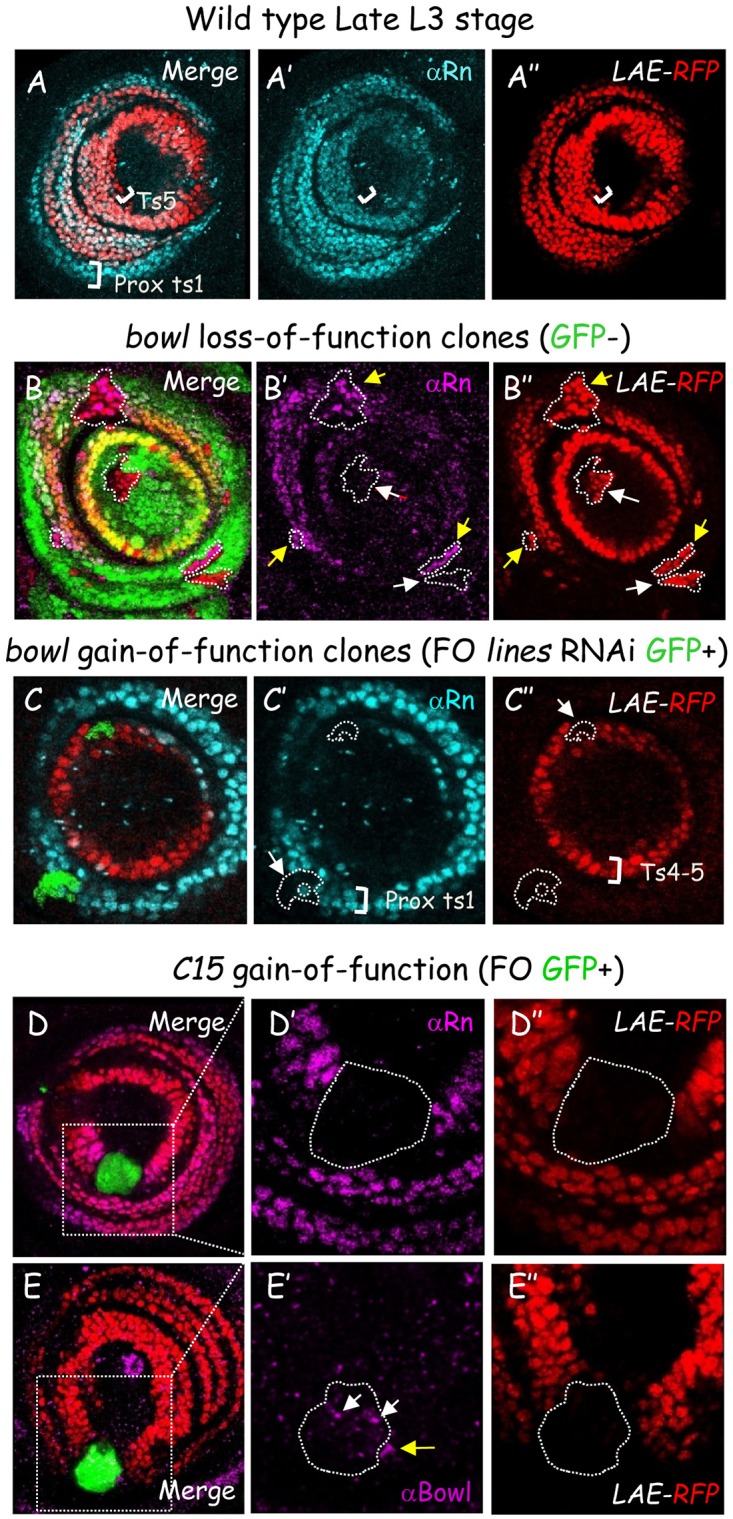
Bowl and C15 activities repress jointly *rotund* expression. (A) Rn protein is detected lately in the emerging tarsus of a wild-type late L3 leg disc expressing *LAE-RFP*. Merged Rn immunostaining (cyan) and RFP fluorescence (red) is shown, as well as each marker in isolation, in (A’) and (A”), respectively. Rn and RFP are expressed in overlapping patterns, with proximalmost and distalmost (see also C’) cells expressing only *rn* or *LAE-RFP*, respectively, as indicated by white brackets. (B-C) The Lines/Bowl pathway cell-autonomously regulates *rn* expression in mosaic late L3 leg discs expressing *LAE-RFP* and harboring either *bowl* null mutant clones (GFP-) (B) or *lines* dsRNA-expressing FO clones (GFP+) (C). Merged GFP (green) fluorescence, Rn immunostaining (magenta) and LAE-driven RFP (red) fluorescence are shown, as well as Rn and RFP markers in isolation, in (B’, C’) and (B”, C”), respectively. Mitotic clones are circled with white dashed lines. In striking contrast to *LAE-RFP* (yellow arrows), *rn* was neither de-repressed in distalmost (pretarsal) nor proximalmost *bowl*^*-/-*^ clones (white arrows). Conversely, Rn and RFP expression were both down-regulated in clones ectopically-stabilizing nuclear Bowl (white arrows). (D-E) Ectopic C15 protein represses *rn* expression, in a Bowl-independent manner, in mosaic late L3 leg discs expressing *LAE-RFP* and harboring FO clones (GFP+) ectopically-expressing C15 homeoprotein. Merged GFP (green) fluorescence, Rn (D) or Bowl (E) immunostaining (magenta) and LAE-driven (RFP) fluorescence are shown. Boxed areas are magnified for each marker in isolation, in (D’, E’) and (D”, E”), respectively, with large FO clones circled with white dashed lines. As for *LAE-RFP*, Rn was fully extinguished in tarsal cells ectopically-expressing C15 protein. Note that nuclear Bowl was autonomously-stabilized in a few *C15* misexpressing cells (white arrows), as well as non-autonomously in neighboring GFP negative cells (yellow arrow), as shown in [8].

First, we sought to examine *rn* expression. To this end, we generated a polyclonal antibody recognizing Rn (see Materials and methods). While *in situ* hybridization indicated transient expression limited from early-mid to mid-late L3 stages [10, 27], nuclear Rn could be detected later on. Indeed, at the late L3 stage Rn and *LAE-RFP* are co-expressed in most tarsal cells (Fig 8A), with Rn extending more proximally other several cell rows, while conversely *LAE-RFP* expression extends more distally (Fig 8A, see brackets).

Second, we asked whether the Bowl/Lines cassette regulates *rn* in leg discs expressing *LAE-RFP* as an internal control. As previously observed for *bab2* (Figs 1F and 7C), Rn was cell-autonomously up-regulated in some *bowl* mutant clones (Fig 8B, yellow arrows). Indeed, distalmost and proximalmost mutant clones only up-regulated *LAE-RFP* (white arrows). Conversely, *rn* was cell-autonomously repressed in Bowl gain-of-function clones (Fig 8C, yellow arrow), obtained through FO expression of dsRNA directed against the dedicated Bowl antagonist *lines*. Altogether these data indicate that *rn* expression in the leg disc is regulated negatively by the Lines/Bowl pathway, as previously shown in the antennal disc [13].

Third, we wondered whether *C15* regulates *rn* expression. Contrary to *bab2* which is first expressed as a distal circular domain [20], onset of *rn* expression occurs at the early-mid L3 stage in the emerging tarsal field in the form of a ring between Dac- and Bar-expressing ring-shaped domains [10]. Rn and C15 proteins are thus never abutting. As previously shown for *bab2* (Fig 3E), clonal analyses revealed that *rn* was autonomously repressed in all C15-misexpressing cells (Fig 8D). As previously described [8], tarsal C15 misexpression stabilized nuclear Bowl in some but not all FO cells (Fig 8E, white arrows), thus ruling out that ectopically-stabilized Bowl mediates Rn extinction in C15+ cells.

Fourth, we asked whether *rn* regulates *Dll*, *bowl* and *C15*. In mosaic leg discs expressing the *LAE-GFP* or *LAE-RFP* construct, no effect on Dll, Bowl and C15 expression could be observed either in *rn* loss- or gain-of-function (S5 Fig and Fig 6D). As seen in our previous work [19], we did observe cell-autonomous *LAE-GFP* down-regulation in *rn*^*-/-*^ clones within ts1-2, but not ts3-5 (S5 Fig, panels A-C). Conversely, clonal Rn overexpression led to cell-autonomous *LAE-RFP* up-regulation (S5D Fig, see white arrow), particularly in *bab2*-expressing ts4-5 cells (n = 12).

Fifth, as previously reported [8], misexpressed C15 protein down-regulated *bowl* expression distally (S6A Fig), while conversely ectopically-stabilized Bowl protein did not affect *C15* expression (S6B Fig). Importantly, *bowl* gain-of-function clones (through *lines* down-regulation) leading to *LAE-RFP* repression (Fig 1G), never induced *C15* up-regulation (S6C Fig), confirming that *LAE-RFP* repression by Bowl stabilization within the tarsal cells occurs independently of C15 TF activity.

Lastly, though the distal selector gene *Dll* is required directly or indirectly for expression of *bowl*, *C15* and *rn* [5, 12, 28–30], none was upregulated in mosaic leg discs misexpressing Dll activator (S6D and S6E Fig and Fig 6C) and none affected in turn *Dll* expression in clonal loss- or gain-of-function (see S5A Fig for *rn* as well as S7A and S7C Fig for *bowl* and *C15*, respectively). While as expected (see Fig 1F) *LAE-RFP* was up-regulated in *bowl* mutant clones (S7A Fig), its re-activation occurred only in *Dll*-expressing cells, either distally or proximally (compare white and yellow arrows, respectively). The observation that *LAE-RFP* could only be de-repressed in Dll+ cells is consistent with a critical activating role of the Dll TF in *bab2* expression throughout limb development.

In conclusion, these results indicate that *rn* is also a repressed target of Bowl and C15, drawing new connections within the gene regulatory network governing distal leg development.

## Discussion

The *bric-à-brac2* expression pattern in the developing leg is dynamic [17–19]. EGFR and Notch pathways have been proposed to regulate *bab2* expression in a timely fashion to restrict precisely its expression domain in the distal leg [8, 16]. We previously showed that the Distal-less HD and Rotund ZF transcription factors are central to activating *bab2* in the developing distal leg, by binding directly a single tissue-specific enhancer, the LAE [19]. Here, we identified two transcriptional repressors, the C15 HD and the Bowl ZF proteins, that likewise act though LAE by repressing directly *bab2* in pretarsal (Bowl and C15) as well as in proximal ts1 and distal ts5 cells (Bowl), to delimit precisely its final tarsal expression domain within the Dll-expressing cells. Moreover, we found that C15 does not require regular partnership with Aristaless for *bab2* repression. Instead, C15 interacts functionally with Distal-less, engaging a direct molecular partnership through their DNA-binding homeodomain and competing each other for neighboring binding sites within LAE. Altogether our data allow us to propose an updated model for leg-specific *bab2* regulation, taking into account repressive cell-signaling inputs from EGFR and Notch pathways.

### The Lines/Bowl pathway shapes *bab2* expression pattern in the developing tarsus

Repressive *bowl* activity has been previously proposed to mediate graded *bab2* extinction in response to N signaling, in distal ts5 and in proximal ts1 cells [13, 16]. As for other tissues, *bowl* activity in the leg disc is down-regulated by *lines* activity [13, 15, 31], presumably through a direct physical association between the Bowl and Lines proteins [15]. In the present study, we provided several lines of evidence that the Lines/Bowl pathway regulates *bab2* expression in developing distal leg cells through direct interaction of the Bowl ZF domain with conserved Odd family binding motifs within CR2 [21, 22, 32]. First, both *bab2* expression and *LAE-RFP* reporter activity are cell-autonomously de-repressed in *bowl* mutant clones. Second, ectopic nuclear stabilization of Bowl TF (through *lines* down-regulation) is sufficient for cell-autonomous *bab2* (*LAE-RFP*) down-regulation in developing tarsal cells. Third, recombinant Bowl ZF interacts directly with conserved LAE motifs, in a sequence-specific manner. Lastly, these binding sites are relevant *in vivo*, since ectopically stabilized Bowl protein is unable to extinguish a *bab2* reporter gene mutant for the two biochemically-defined binding sites, confirming their critical role in mediating LAE enhancer repression.

As predicted from a bacterial one-hybrid assay [21], Bowl and Odd proteins interact *in vitro* with highly similar DNA-binding motifs [22] (this study). It is worth noting that *bab2* is the first direct target gene identified so far for the Bowl TF. Although the near-palindromic CR2 cognate site is critical, a second one situated approximately 100 bp away is required specifically for full repression of the LAE enhancer in proximal leg tissues (Fig 2C). How the two Bowl ZF-binding sites cooperate in proximal ts1 cells remains to be deciphered. Interestingly, cooperation between distant cognate sites has been suggested for transcriptional targets of the related Odd TF during embryogenesis [22].

### C15 acts independently of Al to mediate repressive EGFR signaling input on *bab2* expression

Initially expressed within a broad, distal circular domain at the early L3 stage, *bab2* is extinguished in the developing pretarsus in response to EGFR signaling activation at early-mid L3 stage [5]. Among known EGFR signaling targets [3], we provided several lines of evidence that the C15 transcription factor is directly repressing *bab2* and do so independently of its regular partner Al. First, C15 protein behaves as a strong LAE interactor in yeast cells. Second, C15 homeodomain interacts strongly *in vitro* with dedicated binding sites within the critical CR1 LAE sequence, without synergistic effect of Al (contrary to *Bar* enhancer sequences). Third, both *bab2* expression and *LAE-RFP* reporter activity are de-repressed distally in leg discs deficient for *C15* activity. Fourth, ectopic expression of the C15 homeoprotein is sufficient to extinguish cell-autonomously *bab2* (and LAE reporter activity) in tarsal cells, even in Al deficient cells. Altogether our data suggest that *Bar* regulation by the C15-Al heterodimer does not reflect a rule. Characterization of other direct target genes, among candidates such as *rn* (this work) and *Dl* [8], will indicate to what extent C15 TF is acting independently of Al.

### C15 and Dll interact physically and compete for LAE binding

Within the essential CR1 sequence, C15 interacts strongly with an A/T-rich motif (S6) adjacent to the critical TAAT-rich Dll homeoprotein interacting DBS (Fig 4), which is not required for efficient C15 binding to its neighboring site S6. This proximity has prompted us to examine whether C15 and Dll are able to bind simultaneously to CR1 sequences encompassing both sites. Surprisingly, in EMSA experiments, simultaneous addition of both homeoproteins never revealed C15-Dll heterodimeric binding. We showed that C15 is able to compete with Dll upon binding to CR1 sequences, in a dose-sensitive manner (Fig 6A). Moreover, we found that C15, but not Al, interacts physically with Dll through their homeodomain (Fig 6B), providing a molecular mechanism for their antagonistic DNA binding to LAE sequences. Thus, C15 engages intermolecular interactions with both Al and Dll, through their related DNA-binding domain but with opposite outcomes: cooperative binding to specific DNA sequence (C15-Al) versus competition for LAE binding (C15-Dll). Further studies will determine relevant mechanism(s) of *bab2* repression by C15 among at least three non-exclusive possibilities: (1) Dll-independent C15 repressive activity through direct binding on its own cognate sites within LAE (presumably through interaction with co-repressors, see below); (2) direct C15 binding to the S6 CR1 sequence sterically hindering Dll binding to its own adjacent cognate site (DBS); and (3) heterodimerization with Dll, inhibiting its direct interaction with LAE or changing its global DNA binding specificity, independently of C15 DNA binding per se. Whatever the mechanism(s) involved, our data suggest that C15 and Dll compete functionally *in vivo* in differentially regulating LAE activity, i.e. repression versus activation (Fig 6C). Given that both C15 and Dll (as well as Al) are co-expressed in pretarsal cells, we speculate that the relative amounts of C15 and Dll homeoproteins are critical for full repression in the distal leg. Interestingly, mammalian homologs of Dll form heterodimers with repressive Msx-type homeoproteins, through their respective HDs, with mutually antagonistic interactions *in vitro* and *in-vivo* [33], indicating an evolutionarily-conserved propensity of Dll-type HDs in assembling heterodimeric complexes with repressive homeoproteins.

We thus propose that at early-mid L3 stage, before *C15* activation, Dll expressed throughout the distal territory allows *bab2* induction as a distal circular domain, by binding the DBS within LAE. At the onset of *C15* expression in response to EGFR signaling, increasing amount of C15 protein competes with Dll activator in pretarsal cells, thus resolving *bab2* expression pattern as a single ring of cells at the mid L3 stage.

As stated above, in addition to interacting and/or competing with activating Dll on binding CR1 sequences, C15 might repress LAE activity on its own. Consistently, C15 harbors an eh1-type repressor domain that can interact with the Groucho (Gro) co-repressor [34]. Additionally, we showed that Bowl must function together with C15 to fully repress LAE-activity (Fig 7C). It is noteworthy that Bowl also harbors an eh1 repressive motif [35]. As for C15 (above), Bowl ZF protein is therefore predicted to interact with the Gro co-repressor and both repressive transcription factors may extinguish *bab2* through the same molecular mechanisms, delimiting the tarsal expression domain in a timely manner. Future work should determine whether the C15 transcription factor functions together with the Gro protein to extinguish *bab2* in the developing pretarsal primordium.

### A model for molecular integration of multiple cell signaling inputs by the LAE CRM

**Fig 9:**
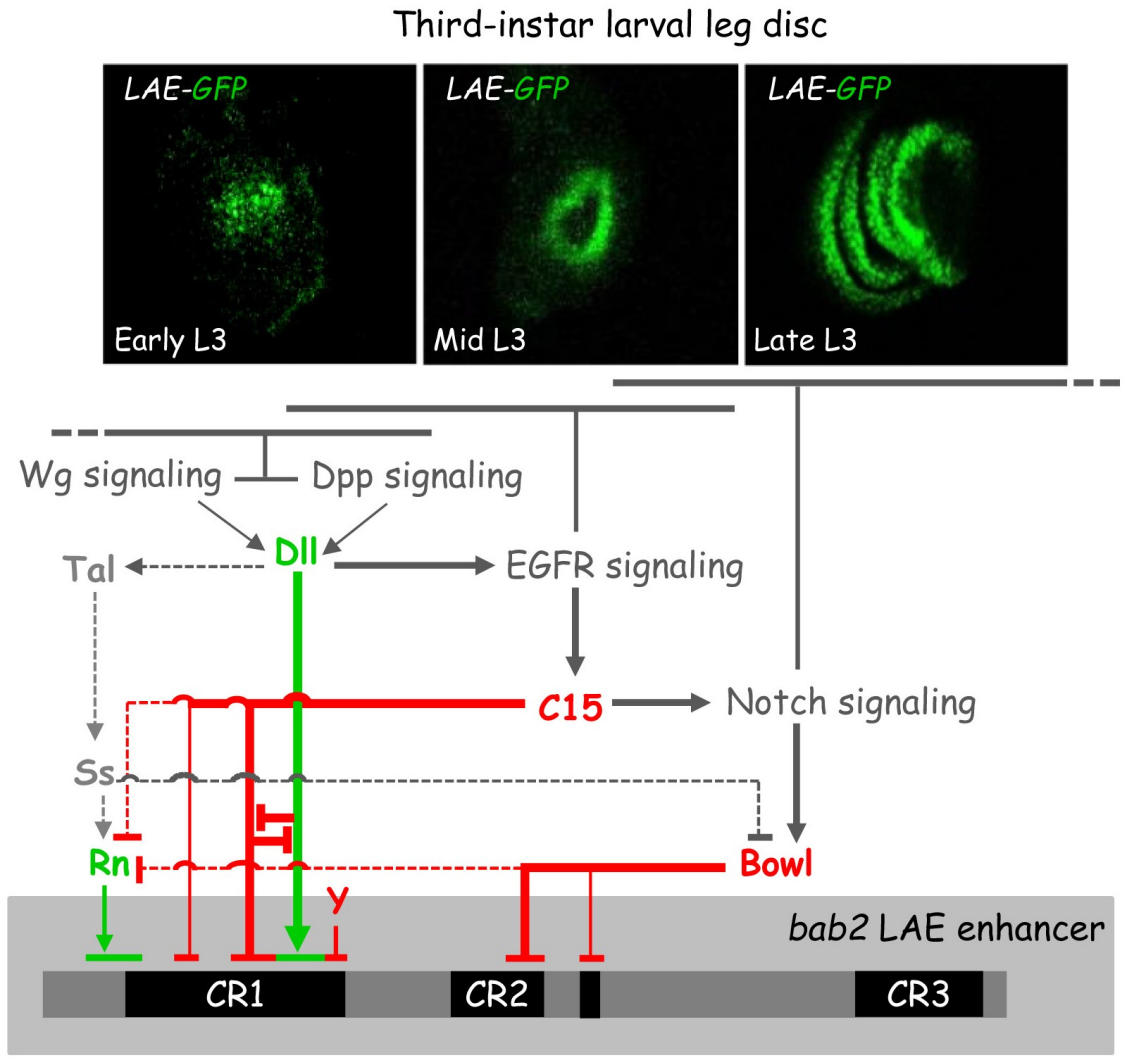
An updated model for limb-specific *bab2* regulation: The LAE enhancer integrates multiple cell-signaling pathways. See main text for details.

Taken together with our previous work [19], we propose an updated model for LAE regulatory activity in the developing distal leg (Fig 9). The tissue-specificity of the LAE enhancer is ensured by its CR1-2 region, while its 3’-end moiety, containing CR3, is mainly required for transcriptional enhancer strength. Situated downstream of the Wg and Dpp signaling inputs, the Distal-less homeodomain TF is required to induce and maintain overall *bab2* expression within the developing tarsus, from L3 to adult stages, by acting through CR1 [19]. Given that Dll is expressed early on during larval development, well before *bab2* expression onset as a circular distal domain, another transcriptional activator functioning at early-mid L3 stage remains to be identified. This factor may operate through activating CR1 sequences overlapping the C15 binding sites (S4B Fig).

From early-mid to mid-late L3 stages, in response to Tal signaling induced by EGFR together with Dll inputs and via *spineless*-mediated activation [10, 29], Rn is activated in the ts1-3 primordia and up-regulates LAE activity in the proximalmost *bab2*-expressing cells. Given the dynamic activity of Bowl protein during tarsal growth, de Celis and Bray [16] anticipated that a transiently-expressed *bab2* activator must counteract repressive Bowl TF in developing tarsal cells. Our previous work indicates that the Rn ZF protein could be this factor [19]. The Rn transcriptional activator is indeed expressed at the right time and in the proper cells, counteracting directly ongoing repressive Bowl on LAE enhancer activity. Interestingly, dynamic *bowl* activity is under the negative control of the transiently-accumulated Spineless (Ss) bHLH-PAS TF [16], which itself is positively required for *rn* activation [10]. Lastly, Bowl activity represses *rn* expression (this work), suggesting that Ss may in fact induce *rn* by counteracting Bowl repression.

Concomitant to the onset of Rn activity in proximal tarsal cells, and in response to EGFR signaling at early-mid L3 stage, C15 is activated and alone (i.e. without Al) extinguishes *bab2* in the pretarsal primordium, presumably in a dose-dependent manner (see above), through direct competitive interactions with Dll (this work). At the same time, *C15* cell-autonomously down-regulates *Dl* in the developing pretarsal cells, allowing *bowl* up-regulation in response to activated N signaling at the tarsus/pretarsus boundary [8]. Thus, *C15* is required non-cell-autonomously for *bowl* expression in developing ts5 [8]. There, the Bowl TF in turn represses LAE enhancer activity (through its CR2 cognate site) to reshape the distalmost *bab2*-expressing ring during mid to late L3 stages. Lastly, at the late L3 stage and beyond up to the adult stage, graded *bab2* expression is observed in each tarsal segment [17]. To complete our model regarding the molecular inputs controlling LAE enhancer activity, the previously-identified CR1-binding inter-ring repressive activity [19] (Factor Y in Fig 9) and the *bab2* activator acting during the early L3 stage (X) remain to be characterized.

Finally, this work also shed light to new epistatic relationships between *bab2* regulators within the leg genetic cascade. In addition to *bab2*, we showed that *C15* and *bowl* regulate also *rotund*. Nevertheless, direct interactions with *rn* enhancer sequences remain to be established. It would be interesting to determine whether C15 (i) functions jointly with Bowl and/or (ii) antagonizes Dll activity in repressing other target genes.

## Materials and methods

### Fly stocks, culture and genetic manipulations

*Drosophila* lines were grown on standard yeast extract-sucrose medium. The *vasa*-PhiC31 ZH2A *attP* stock (kindly provided by F. Karch) was used to generate the mutant *LAE-GFP* reporter lines, as previously described [19]. *LAE-RFP* constructs were generated by insertions on both the ZH2A (X chromosome) and ZH86Fb (third chromosome) *attP* landing platforms, and they display identical expression patterns [19]. *UAS-C15*, *UAS-Dll* and *UAS-Rn* stocks were obtained from T. Kojima, S. Cohen and the Bloomington stock Center (#7403), respectively. *C15*^*2*^/TM6B, *Tb*^*1*^ stock was kindly provided by G. Campbell. Mutant mitotic clones for null alleles of *bowl*, *C15* and *rotund* were generated with the following genotypes: *y w LAEwt-RFP*; *DllGal4*^*EM2012*^ *UAS*-*Flp / +*; *Ub-GFP FRT40A*/*bowl*^*1*^ *FRT40A* (i.e., *bowl* mutant clones are GFP negative; Figs 1F, 7C and 8B and S7A Fig), *y w LAEwt-RFP*; *DllGal4*^*EM2012*^ *UAS*-*Flp / +; FRT82B/FRT82B Ub-GFP C15*^*2*^ (i.e., *C15* mutant clones harbor two *Ub-GFP* copies; Fig 7A and 7B) or *y w LAEwt-GFP* (or *LAEm3-GFP*); *DllGal4*^*EM2012*^ *UAS*-*Flp / +; Ub-RFP FRT82B / FRT82B C15*^*2*^ and *y w LAEwt-GFP*; *DllGal4*^*EM2012*^ *UAS*-*Flp / +; Ub-RFP FRT82B/FRT82B rn*^*16*^ (i.e., *C15* or *rn* mutant clones are marked by the absence of RFP fluorescence; Fig 7D and S5A–S5C and S7C Figs). *UAS-dsRNA* stocks used to obtain RNAi against *lines* (#40939) or *al* (#26747) were obtained from the Bloomington stock Center. “Flip-out” mitotic clones over-expressing dsRNA against *lines* were generated by 40 mn heat shocks at 38°C, in early second- to early-mid L3 larvae of genotypes: *y w LAEwt-RFP hsFlp*; *UAS-dsRNAlines / Pact>y+>Gal4 UAS-GFP* (i.e., FO clones express GFP in Fig 1G) and *y w LAEwt-GFP* (or *LAEm3-GFP*) *hsFlp*; *UAS-dsRNAlines / Pact>y+>Gal4 UAS-RFP* (i.e., Lines-deficient FO clones express RFP in Figs 2F, 2G and 8C and S6B, S6C and S7B Figs). FO clones overexpressing C15, Rn or Dll were generated by 40 mn heat shocks at 38°C, in mid second to early-mid L3 larvae of genotypes: *y w LAEwt-RFP hsFlp*; *UAS-C15 / Pact>y+>Gal4 UAS-GFP; (+/-UAS-dsRNAal / +)* (i.e., FO clones express GFP; Figs 3E, 5C, 5D, 8D and 8E and S6A and S7D Figs), *y w LAEwt-RFP hsFlp*; *UAS-Rn / Pact>y+>Gal4 UAS-GFP* (i.e., FO clones express GFP; Fig 6D and S5D and S5E Fig), and *y w LAEwt-RFP hsFlp*; *Pact>y+>Gal4 UAS-GFP / +; UAS Dll / +* (i.e., FO clones express GFP: Fig 6C and S6D and S6E Fig), respectively.

### Reporter construction and mutagenesis

LAE fragments were cloned into pBP-S3aG vector as previously described [19]. pLAE-GFP or -RFP site-directed mutagenesis was performed by PCR, using the overlap extension method [36]. All plasmid constructs used in this work were sequence verified. Mutated LAE reporters were all inserted on the same ZH2A *attP* landing platform.

### Yeast one hybrid assay

The Y1H screen was performed as described in [23]. LAE and mini-LAE DNA baits were amplified by PCR and cloned into pENTRY-5’ using standard restriction-ligation techniques. The sequence-verified LAE baits were sub-cloned into the Y1H-compatible pMW2 (*HIS3*) vector by Gateway LR cloning. LAE destination clones were integrated in Y1H-YM4271 yeast strain using lithium acetate-polyethylene glycol transformation followed by selection on a synthetic complete (SC) (-His) medium plate.

### Immuno-histochemistry and microscopy

Leg discs were dissected from wandering L3 larvae. Indirect immuno-fluorescence was carried out as previously described [19] using a LEICA TCS SP5 or SP8 confocal microscope. Rat anti-Bab2 [18], rabbit anti-Bowl [16], rabbit anti-C15 [9] and rat anti-Al [8] antibodies, kindly provided by F. Laski, S. Bray, T. Kojima and G. Campbell, respectively, were used at 1/2000, 1/1000, 1/200 and 1/1000, respectively. Guinea pig antibody raised against Roe (an isoform encoded by the *rotund*/*roe* locus, see below) was prepared by Eurogentec, from a full-length GST-Roe fusion (a kind gift from D. del Alamo) [37] extracted from *E*. *coli* and enriched by affinity chromatography. Note that (i) anti-Roe cross-reacts with Rn, because both proteins share a large common C-terminal region (with the ZF domain) and (ii) Roe protein isoform is not expressed in the leg [27]. Anti-Roe/Rn sera from terminal bleeds was used for immunocytology without purification at a 1/500 dilution.

### Recombinant proteins

The Open Reading Frame (ORF) encompassing the C15 HD-encoding sequence (codons 192 to 292) was amplified by PCR, using as a template a full length (FL) cDNA insert (IP08859), obtained from the *Drosophila* Genomic Resource Center (DGRC), and inserted as an *Eco*RI/*Xho*I fragment into pGex4T, to yield the pGexC15^HD^ construct. Similarly, Dll-encoding sequences (full-length ORF or codons 120–188) were amplified by PCR, using as a template a full ORF cDNA insert [23], and inserted as *Eco*RI/*Xho*I fragments into pGex4T, to yield the pGexDll^FL^ and pGexDll^HD^ constructs. Non-fused GST (produced from empty pGex4T), GST-C15^HD^ and both GST-Dll proteins were all expressed in BL21 [DE3] cells (Novagen), from which soluble forms were purified on glutathione-Sepharose beads (GE Healthcare) and then isolated through elution with free glutathione. Purified proteins were used in EMSA, as described in [26]. Given that FL Bowl protein appeared toxic when expressed in *E*. *coli* cells, ZF domain encoding *bowl* ORF (codons 220 to 382) was amplified by PCR, using as template a FL cDNA clone (a kind gift from Sarah Bray), and inserted as an *Eco*RI/*Xho*I fragment into pGex4T, to yield the pGexBowl^ZF^ construct. The GST-Bowl^ZF^ fusion was expressed in BL21 [DE3] cells, from which soluble forms were purified by glutathione-Sepharose affinity. For control EMSA and pull-down experiments, transformation with empty pGex4T was also performed, to produce and then purify soluble non-fused GST.

### Electrophoretic mobility-shift assay

EMSA experiments with Bowl and HD proteins were performed as described in [22] and [26], respectively, using purified GST fusion proteins (above) and/or *in vitro* translated Al HD (see below). Protein-DNA complexes were separated by electrophoresis on 6% native polyacrylamide gels and revealed by PhosphoImager detection. DNA Probes (see Figs 2A and 4B for sequences) were made from annealed synthetic oligonucleotides and 5’end-labeled with [^32^P]dCTP using Klenow DNA polymerase, as previously described [19]. However, a single labeled dCTP (instead of 8 for the LAE-derived probes) was incorporated into the Odd-binding probe, as described in [22]. The BarEnh probe is described in [26].

### GST-pull down assay

Pull down was down as previously described [38], in duplicate. ^35^S protein probes (C15^HD^, Dll^HD^ and Al^HD^) were obtained from coupled *in vitro* transcription/translation (TNT with rabbit reticulocyte extracts; Promega Inc.) from DNA matrices generated by PCR, using 5’ oligonucleotides comprising each a T7 RNA polymerase promoter and a translation initiating ATG within an optimal Kozak context (sequences of oligonucleotides are available upon request). Radiolabeled C15 and Dll HDs (amino acids 192–292 and 120–188, respectively) were produced from full-length cDNA clones (described above). *In vitro* translated Al HD (amino acids 86–156) was produced from a full-length cDNA clone (RE68460), obtained from the *Drosophila* Genomic Resource Center (DGRC).

### LAE sequence identification and alignment

*bric-à-brac* locus sequences were recovered from dipteran genomic sequences and aligned as previously described [19].

## Supporting information

S1 Fig Co-localization of Bowl protein with different *bowl-*unresponsive LAE reporters. (A-B) Late L3 leg discs (distalmost confocal views) expressing either the *LAEwt-GFP* (A) or *LAEΔ2-GFP* (B) reporter. Merged GFP fluorescence (green) and Bowl immunostaining (magenta) are shown. Boxed areas are magnified for the merged markers in (A’) and (B’), as well as GFP expression in isolation in (A”) and (B”), respectively. While *LAEwt-GFP* fluorescence abuts the Bowl+ domain at the ts5-pt boundary, its mutant derivative *Δ*CR2 is specifically up-regulated in many ts5 cells and remained repressed in the raw of *bowl*-expressing pretarsal cells (magenta arrows). (C-D) Late L3 leg discs (distalmost confocal views) expressing the *LAEm3-RFP* reporter. Merged GFP fluorescence (green) and C15 (C) or Bowl (D) immunostaining (magenta) are shown. Boxed areas are magnified for the merged markers in (C’) and (D’), as well as GFP expression in isolation in (C”) and (D”), respectively. Note that *C15*-expressing pretarsal (pt) cells abut GFP-expressing ts4-5 cells [white bracket in (C”)], indicating that Bowl-insensitive *LAEm3-GFP* is still repressed in the developing pretarsus, including in a row of Bowl-expressing cells [white bracket in panel (D”)].
(TIF)

S2 Fig The Bowl ZF binding sites within LAE are conserved among dipterans. (A-B) CR2 (A) and 3’-neighboring region of CR2 (B) sequences of 27 dipterans are aligned. Drosophilidae, *C*. *capitata*, *G*. *morsitans* and *M*. *domestica* LAE-like sequences were identified though BLAST analyses using the Trace archive nucleotide blast server at NCBI (http://blast.ncbi.nlm.nih.gov/Blast.cgi) and were aligned with the *D*. *melanogaster* LAE sequence using MAFFT (http://mafft.cbrc.jp/alignment/server/index.html). Homology shading was performed using BoxShade (http://www.ch.embnet.org/software/BOX_form.html). Bowl BS positions are indicated by blue dashed boxes. The near-palindromic site within CR2 is indicated by an orange dashed box.
(TIF)

S3 Fig *C15* is required for distal *bowl* expression. (A-B) Late L3 late discs from wild-type (A) or *C15*^*2*^ homozygous mutant (B) expressing *LAE-RFP*. Merged RFP fluorescence (red) and Bowl immunostaining (magenta) are shown, as well as the latter in isolation, in (A’) and (B’). It is noteworthy that *bowl* expression at the ts5/pretarsal (pt) boundary [yellow arrows in (A) and in (A’)] is no longer detected in the *C15* loss-of-function developing distal leg, while remaining unaffected at the tibial (ti)/ts1 boundary [white arrows in (B) and (B’)].
(TIF)

S4 Fig C15 binding sequences are also required for LAE activation. (A) C15 homeodomain binds distinct A/T-rich motifs within the LAE CR1 sequence. EMSA experiment with retarded complexes obtained from purified GST-C15^HD^ and unfused GST as a negative control. A non-specific retarded complex is indicated by asterisks. See Fig 4B for probed CR1 sequences. (B) The C15 repressor binding sites within CR1 are critical for LAE activation *in vivo*. GFP (green) and RFP (red) fluorescence are shown for late L3 leg discs expressing *LAEwt-GFP* together with wild-type (upper panels) or mutated (lower panels) *LAE-RFP* constructs. The *F2LAE*^*S3+4+6m*^ construct mutated for both C15 binding sites within CR1 (identical to the CR1^S3+4+6m^ DNA probe depicted in B; lane 10) does not expressed RFP within the leg discs, suggesting that the S3-4 and S6 motifs encompass activating sequences.
(TIF)

S5 Fig *rotund* does not regulate other *bab2* regulators. Mosaic late L3 leg discs expressing *LAE-GFP* (A-C) or *LAE–RFP* (D-E) and harboring either *rotund* null clones (detected by the loss of RFP) (A-C) or FO clones (specifically expressing GFP) overexpressing the Rn protein (D-E). Merged RFP fluorescence (red), GFP fluorescence (green) and Dll (A, D), Bowl (B, E) or C15 (C) immunostaining (magenta) are shown, as well as the latter and GFP or RFP markers in isolation, in (A’-E’) and (A”-E”), respectively. Mitotic clones are circled with white dashed lines. In striking contrast to *LAE-RFP* (specifically affected in ts1-2 rings, white arrows), *Dll*, *bowl* and *C15* expression remained unchanged in *rn* null clones. Similarly, misexpressed Rn TF never detectably affected *Dll*, *bowl* nor C15 (see Fig 6D) expression, while *LAE-RFP* was slightly up-regulated in ts3-5 FO clones (white arrow).
(TIF)

S6 Fig Epistatic relationships among *bab2* regulators. Mosaic late L3 leg discs expressing *LAE-RFP* and harboring FO clones (specifically expressing GFP) ectopically-expressing C15 (A), Bowl (B-C) or Dll (D-E). Merged markers and Bowl, C15 or Rn immunostaining (magenta) are shown, as well as *LAE-RFP* fluorescence in isolation. FO mitotic clones are circled with white dashed lines and some are indicated with white or yellow arrows. Lateral confocal views are shown in (A) and (B) with distalmost pretarsal cells on the left and right side, respectively. Note that ectopically-expressed C15 protein down-regulated *bowl* expression at the pt-ts5 boundary, while the converse was not observed (yellow vs white arrows). As expected *LAE-RFP* was autonomously extinguished in Lines-depleted tarsal FO cells (C”), but C15 protein was never detected there (C’) (yellow arrows), thus discarding the possibility that *C15* misexpression contributes to *LAE-RFP* repression by ectopically-stabilized nuclear Bowl. Lastly, whereas *rn* remained unaffected in all Dll-misexpressing FO clones (E’, yellow arrow), a faint cell-autonomous *LAE-RFP* up-regulation was observed (albeit not in all cells) in some proximalmost clones (E”, yellow arrow), indicating that misexpressed Dll is sufficient to activate the LAE in proximal cells, as shown in C15-expressing distal cells (Fig 6C).
(TIF)

S7 Fig *bowl* and *C15* do not regulate *Dll* expression. Mosaic late L3 leg discs expressing *LAE-RFP* (A, B, D) or *LAE-GFP* (C) and harboring loss- or gain-of-function clones for *bowl* (A, B) or *C15* (C-D). Merged RFP fluorescence (red), GFP fluorescence (green) and Dll immunostaining (magenta) are shown, as well as the Dll and RFP or GFP markers in isolation, in (A’-D’) and (A”-D”), respectively. Mitotic clones are circled with white dashed lines. In striking contrast to *bab2* reporters, *Dll* expression was never detectably affected in any mosaic discs (white arrows). As expected, LAE reporter activity was down-regulated in FO clones misexpressing Bowl and C15 proteins. *LAE-RFP* up-regulation was observed in all *bowl* mutant cells, provided that they are located within the *Dll*-expressing cells (compare A’ and A”, white vs yellow arrows).
(TIF)
