# Supplementary figures and images for: Tissue-specific enhancer repression through molecular integration of cell signaling inputs
Source: PLoS Genet. 2017 Apr 10;13(4):e1006718. doi: 10.1371/journal.pgen.1006718 (PMC5402979; doi:10.1371/journal.pgen.1006718)

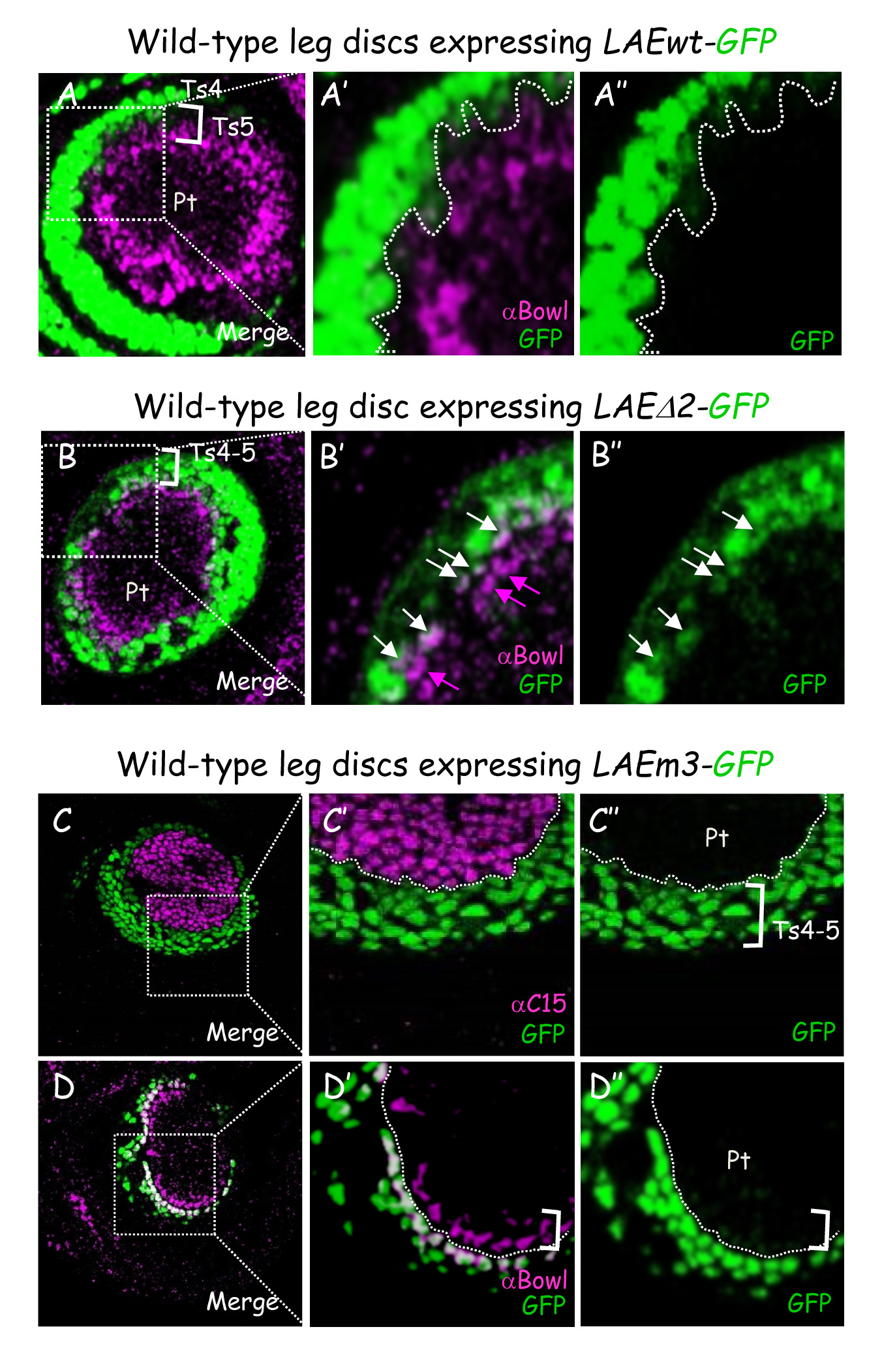

Supplement: S1 Fig — (A-B) Late L3 leg discs (distalmost confocal views) expressing either the LAEwt-GFP (A) or LAEΔ2-GFP (B) reporter. Merged GFP fluorescence (green) and Bowl immunostaining (magenta) are shown. Boxed areas are magnified for the merged markers in (A’) and (B’), as well as GFP expression in isolation in (A”) and (B”), respectively. While LAEwt-GFP fluorescence abuts the Bowl+ domain at the ts5-pt boundary, its mutant derivative ΔCR2 is specifically up-regulated in many ts5 cells and remained repressed in the raw of bowl-expressing pretarsal cells (magenta arrows). (C-D) Late L3 leg discs (distalmost confocal views) expressing the LAEm3-RFP reporter. Merged GFP fluorescence (green) and C15 (C) or Bowl (D) immunostaining (magenta) are shown. Boxed areas are magnified for the merged markers in (C’) and (D’), as well as GFP expression in isolation in (C”) and (D”), respectively. Note that C15-expressing pretarsal (pt) cells abut GFP-expressing ts4-5 cells [white bracket in (C”)], indicating that Bowl-insensitive LAEm3-GFP is still repressed in the developing pretarsus, including in a row of Bowl-expressing cells [white bracket in panel (D”)]. (TIF) [file pgen.1006718.s001.tif]

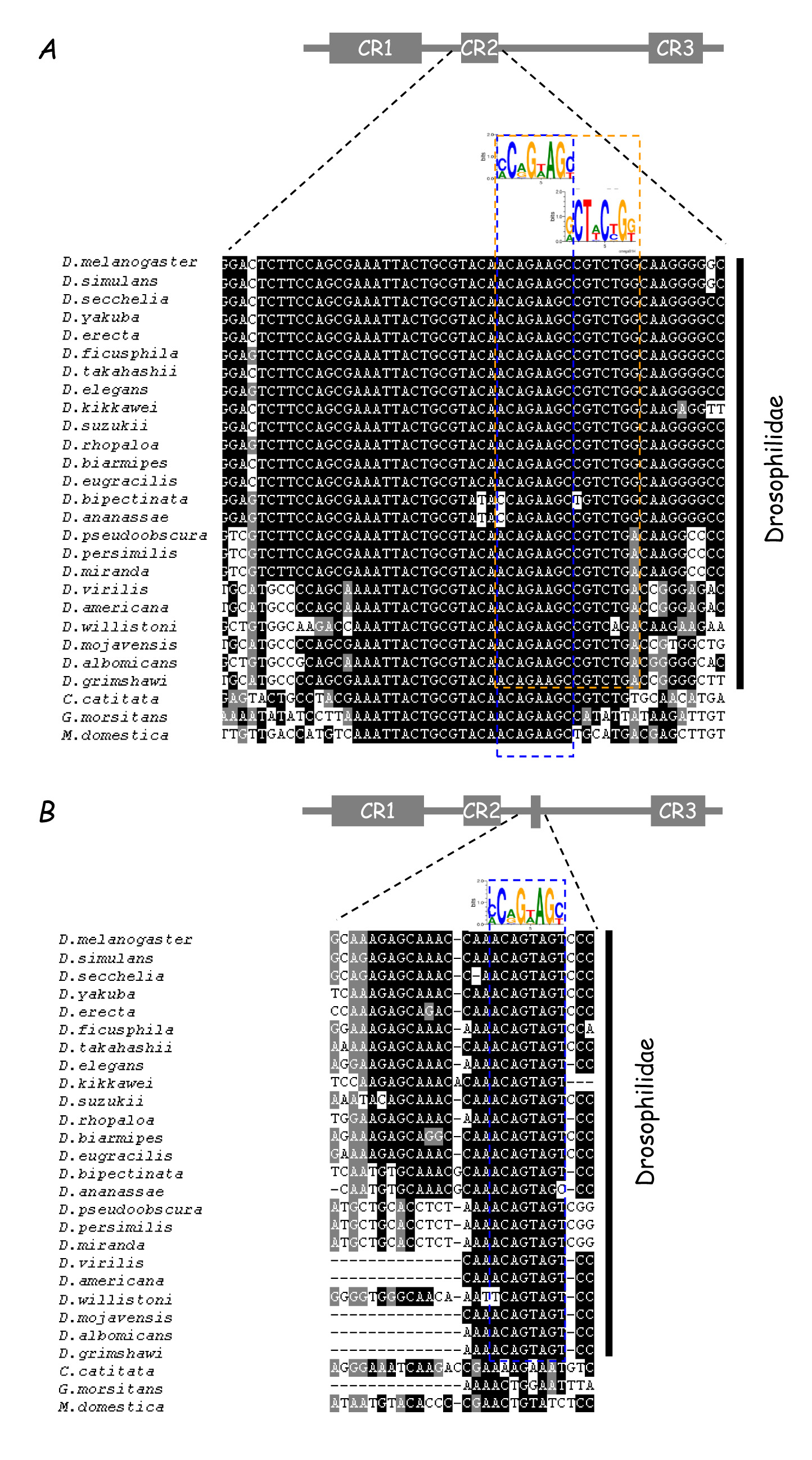

Supplement: S2 Fig — (A-B) CR2 (A) and 3’-neighboring region of CR2 (B) sequences of 27 dipterans are aligned. Drosophilidae, C. capitata, G. morsitans and M. domestica LAE-like sequences were identified though BLAST analyses using the Trace archive nucleotide blast server at NCBI (http://blast.ncbi.nlm.nih.gov/Blast.cgi) and were aligned with the D. melanogaster LAE sequence using MAFFT (http://mafft.cbrc.jp/alignment/server/index.html). Homology shading was performed using BoxShade (http://www.ch.embnet.org/software/BOX_form.html). Bowl BS positions are indicated by blue dashed boxes. The near-palindromic site within CR2 is indicated by an orange dashed box. (TIF) [file pgen.1006718.s002.tif]

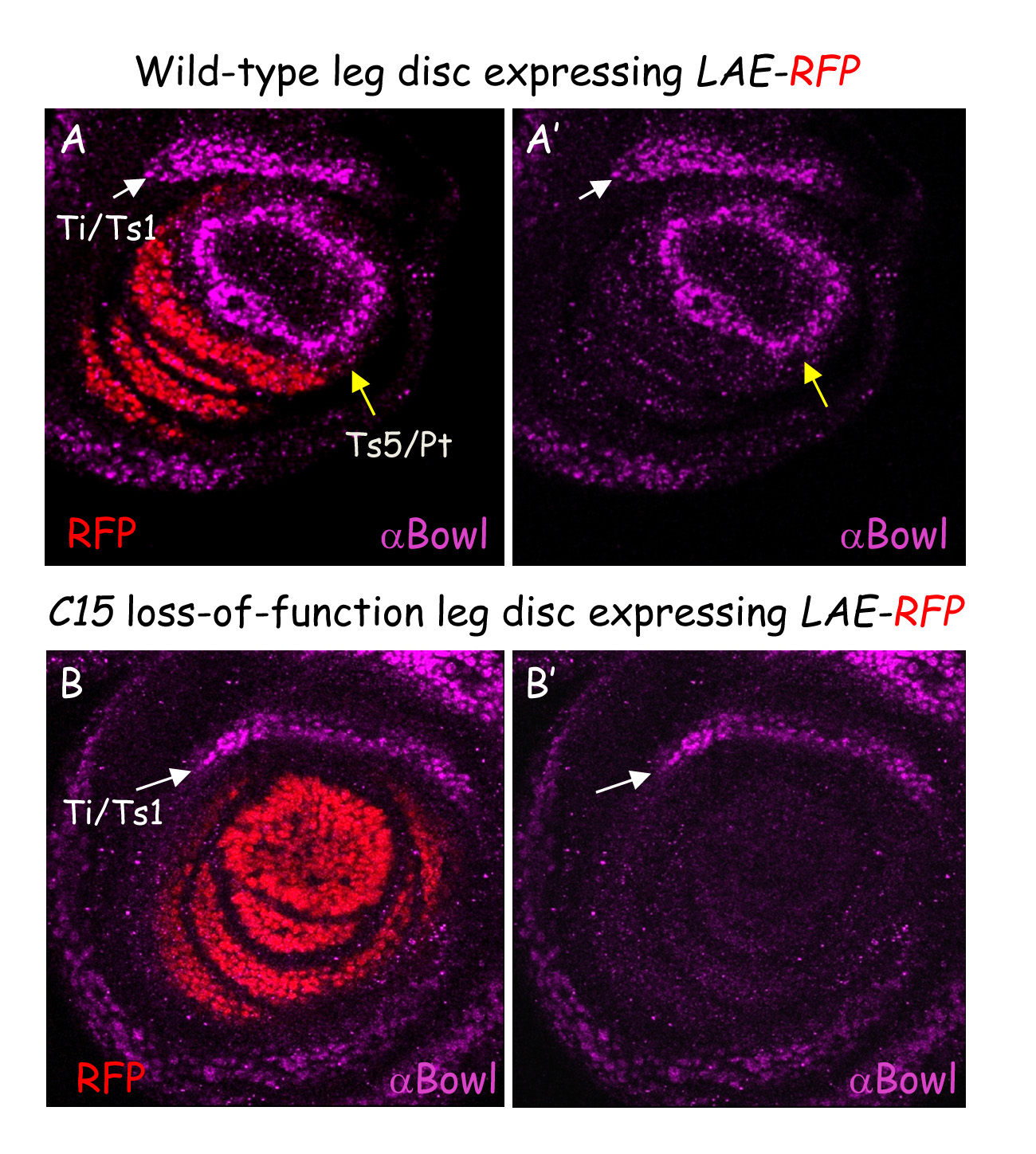

Supplement: S3 Fig — (A-B) Late L3 late discs from wild-type (A) or C152 homozygous mutant (B) expressing LAE-RFP. Merged RFP fluorescence (red) and Bowl immunostaining (magenta) are shown, as well as the latter in isolation, in (A’) and (B’). It is noteworthy that bowl expression at the ts5/pretarsal (pt) boundary [yellow arrows in (A) and in (A’)] is no longer detected in the C15 loss-of-function developing distal leg, while remaining unaffected at the tibial (ti)/ts1 boundary [white arrows in (B) and (B’)]. (TIF) [file pgen.1006718.s003.tif]

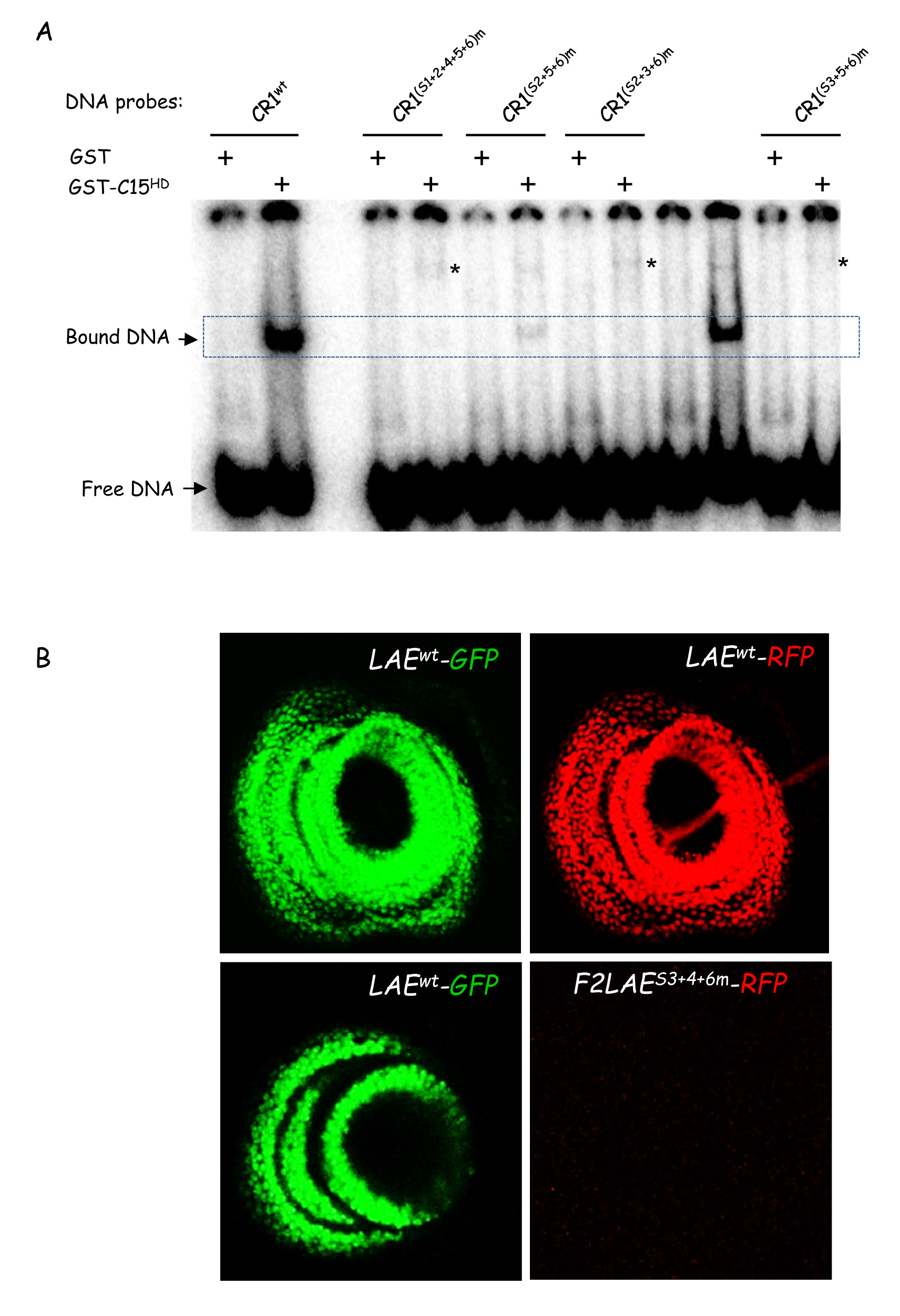

Supplement: S4 Fig — (A) C15 homeodomain binds distinct A/T-rich motifs within the LAE CR1 sequence. EMSA experiment with retarded complexes obtained from purified GST-C15HD and unfused GST as a negative control. A non-specific retarded complex is indicated by asterisks. See Fig 4B for probed CR1 sequences. (B) The C15 repressor binding sites within CR1 are critical for LAE activation in vivo. GFP (green) and RFP (red) fluorescence are shown for late L3 leg discs expressing LAEwt-GFP together with wild-type (upper panels) or mutated (lower panels) LAE-RFP constructs. The F2LAES3+4+6m construct mutated for both C15 binding sites within CR1 (identical to the CR1S3+4+6m DNA probe depicted in B; lane 10) does not expressed RFP within the leg discs, suggesting that the S3-4 and S6 motifs encompass activating sequences. (TIF) [file pgen.1006718.s004.tif]

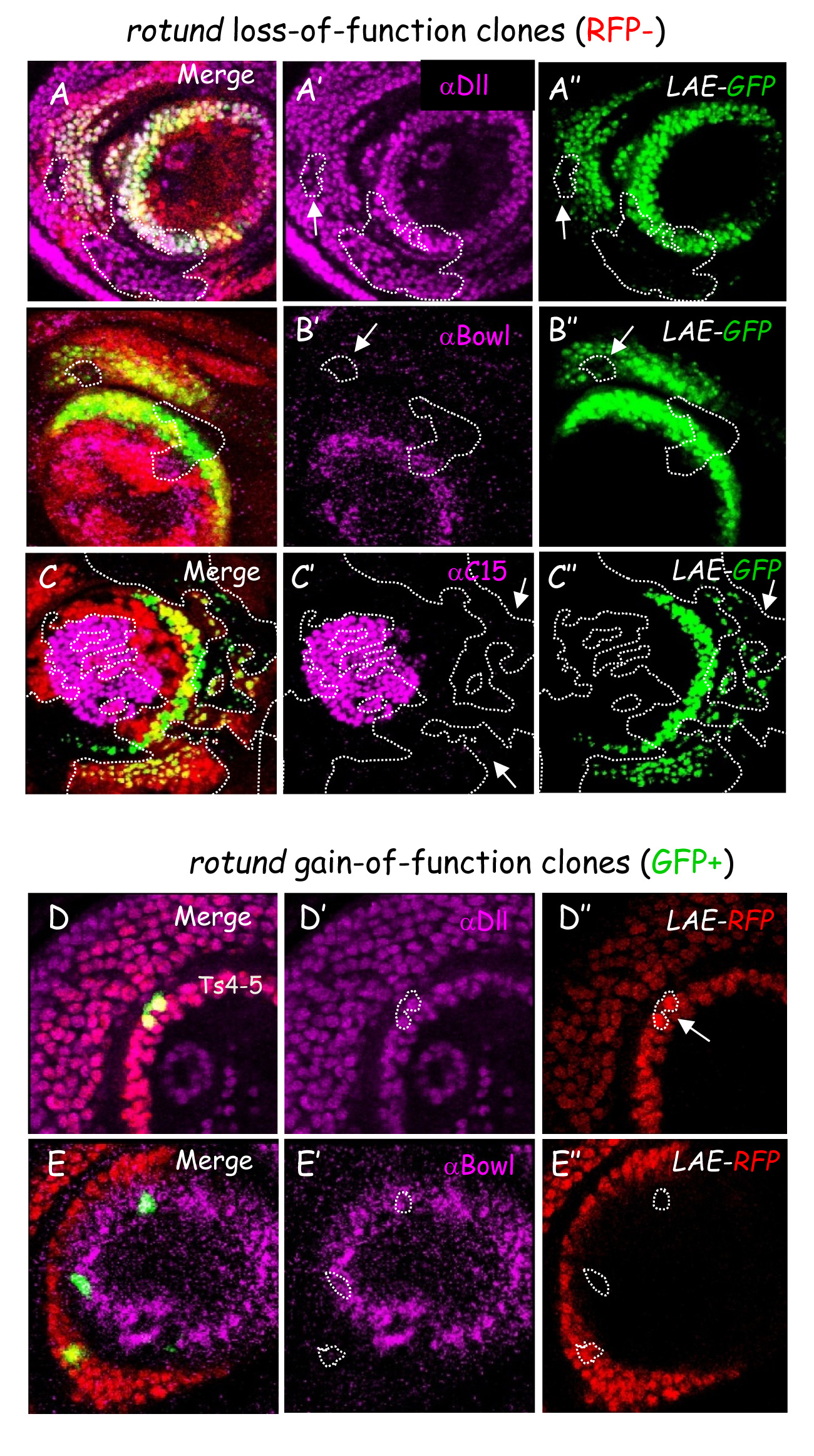

Supplement: S5 Fig — Mosaic late L3 leg discs expressing LAE-GFP (A-C) or LAE–RFP (D-E) and harboring either rotund null clones (detected by the loss of RFP) (A-C) or FO clones (specifically expressing GFP) overexpressing the Rn protein (D-E). Merged RFP fluorescence (red), GFP fluorescence (green) and Dll (A, D), Bowl (B, E) or C15 (C) immunostaining (magenta) are shown, as well as the latter and GFP or RFP markers in isolation, in (A’-E’) and (A”-E”), respectively. Mitotic clones are circled with white dashed lines. In striking contrast to LAE-RFP (specifically affected in ts1-2 rings, white arrows), Dll, bowl and C15 expression remained unchanged in rn null clones. Similarly, misexpressed Rn TF never detectably affected Dll, bowl nor C15 (see Fig 6D) expression, while LAE-RFP was slightly up-regulated in ts3-5 FO clones (white arrow). (TIF) [file pgen.1006718.s005.tif]

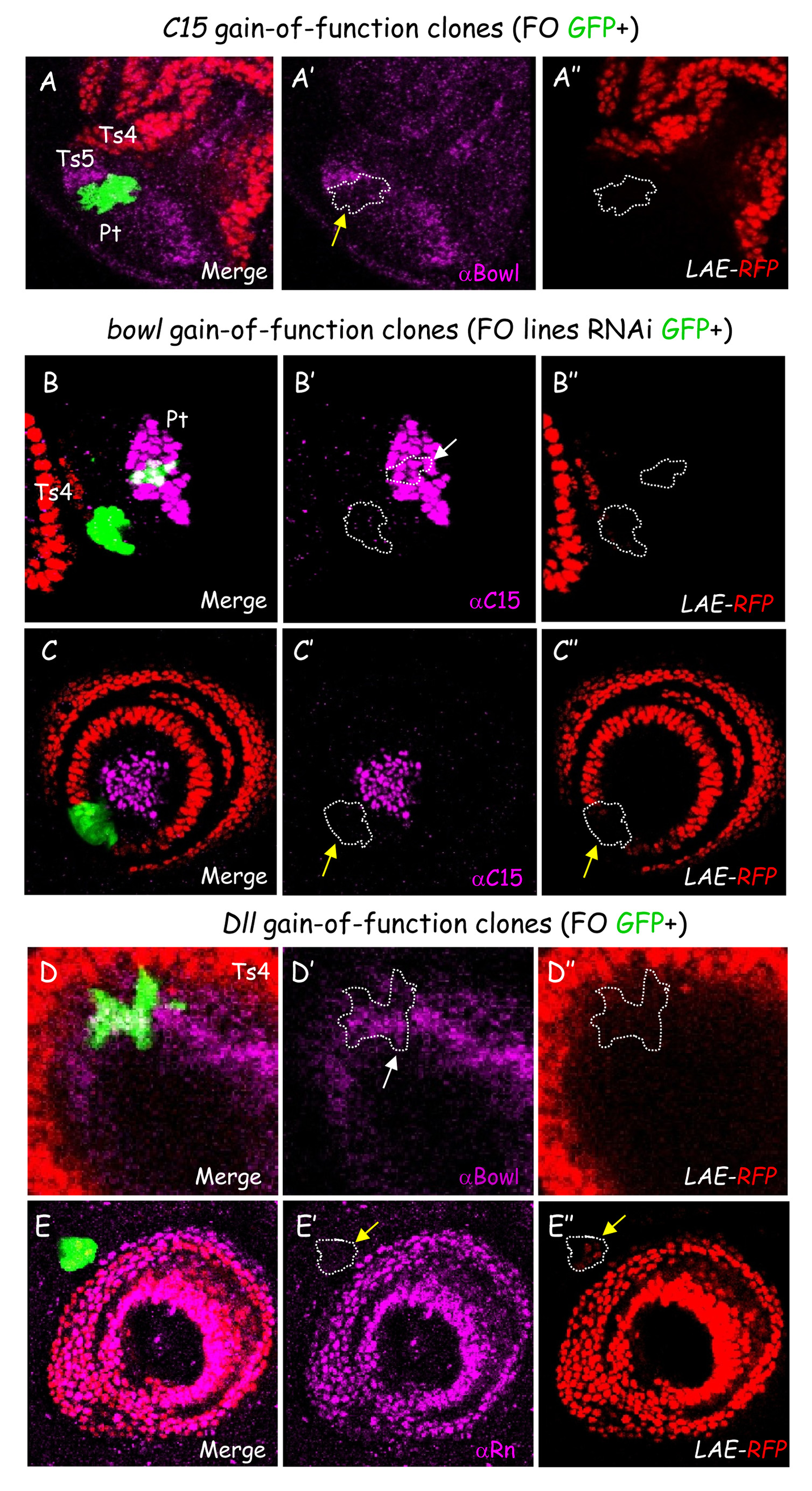

Supplement: S6 Fig — Mosaic late L3 leg discs expressing LAE-RFP and harboring FO clones (specifically expressing GFP) ectopically-expressing C15 (A), Bowl (B-C) or Dll (D-E). Merged markers and Bowl, C15 or Rn immunostaining (magenta) are shown, as well as LAE-RFP fluorescence in isolation. FO mitotic clones are circled with white dashed lines and some are indicated with white or yellow arrows. Lateral confocal views are shown in (A) and (B) with distalmost pretarsal cells on the left and right side, respectively. Note that ectopically-expressed C15 protein down-regulated bowl expression at the pt-ts5 boundary, while the converse was not observed (yellow vs white arrows). As expected LAE-RFP was autonomously extinguished in Lines-depleted tarsal FO cells (C”), but C15 protein was never detected there (C’) (yellow arrows), thus discarding the possibility that C15 misexpression contributes to LAE-RFP repression by ectopically-stabilized nuclear Bowl. Lastly, whereas rn remained unaffected in all Dll-misexpressing FO clones (E’, yellow arrow), a faint cell-autonomous LAE-RFP up-regulation was observed (albeit not in all cells) in some proximalmost clones (E”, yellow arrow), indicating that misexpressed Dll is sufficient to activate the LAE in proximal cells, as shown in C15-expressing distal cells (Fig 6C). (TIF) [file pgen.1006718.s006.tif]

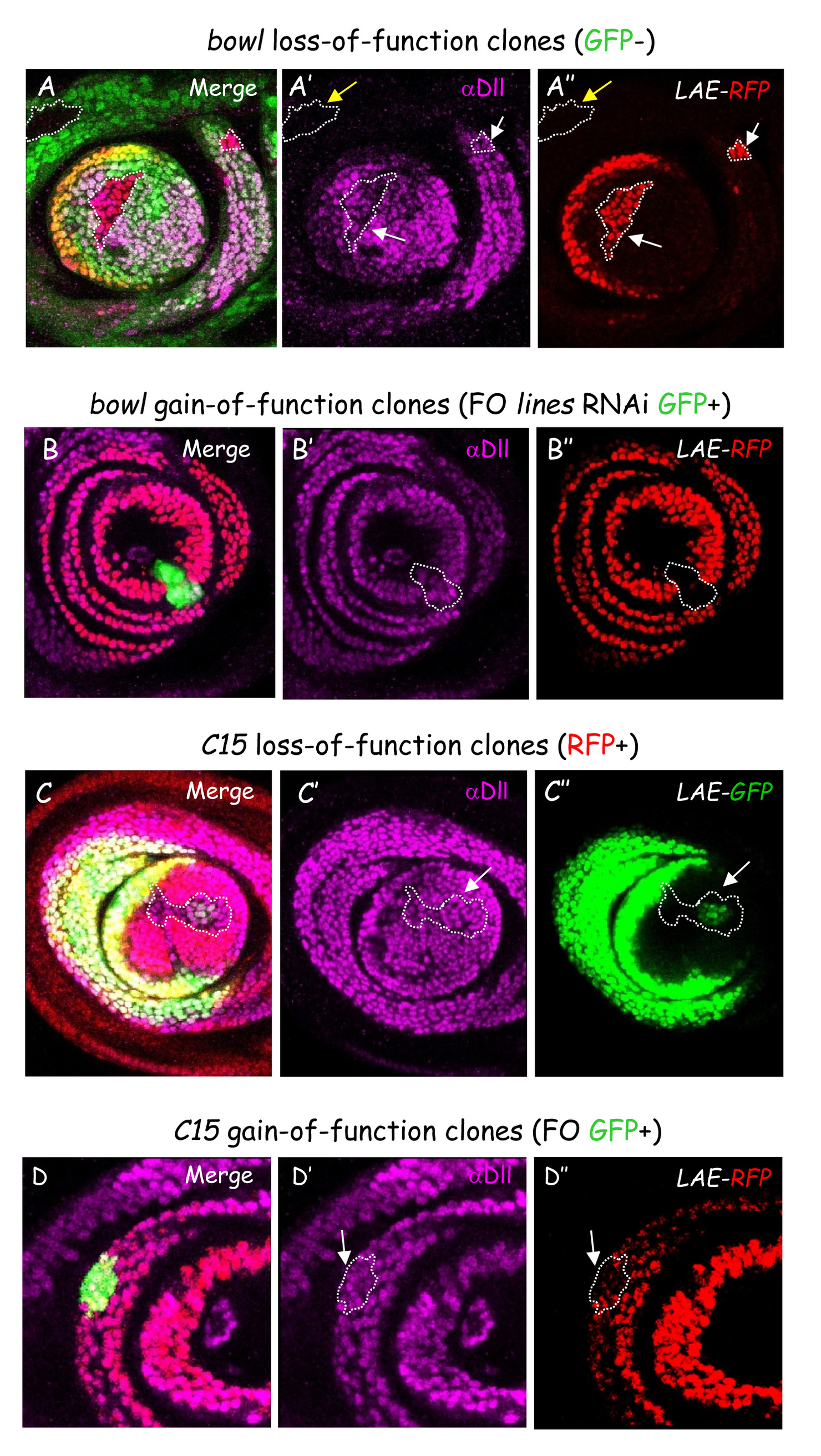

Supplement: S7 Fig — Mosaic late L3 leg discs expressing LAE-RFP (A, B, D) or LAE-GFP (C) and harboring loss- or gain-of-function clones for bowl (A, B) or C15 (C-D). Merged RFP fluorescence (red), GFP fluorescence (green) and Dll immunostaining (magenta) are shown, as well as the Dll and RFP or GFP markers in isolation, in (A’-D’) and (A”-D”), respectively. Mitotic clones are circled with white dashed lines. In striking contrast to bab2 reporters, Dll expression was never detectably affected in any mosaic discs (white arrows). As expected, LAE reporter activity was down-regulated in FO clones misexpressing Bowl and C15 proteins. LAE-RFP up-regulation was observed in all bowl mutant cells, provided that they are located within the Dll-expressing cells (compare A’ and A”, white vs yellow arrows). (TIF) [file pgen.1006718.s007.tif]
